# Unprecedented bacterial community richness in soybean nodules vary with cultivar and water status

**DOI:** 10.1186/s40168-019-0676-8

**Published:** 2019-04-16

**Authors:** Hazem Sharaf, Richard R. Rodrigues, Jinyoung Moon, Bo Zhang, Kerri Mills, Mark A. Williams

**Affiliations:** 1 Interdisciplinary PhD Program in Genetics, Bioinformatics, and Computational Biology, Virginia Polytechnic Institute and State University, Blacksburg, VA USA; 2 School of Plant and Environmental Sciences, Virginia Polytechnic Institute and State University, Blacksburg, VA USA; 3 Present address: Department of Pharmaceutical Sciences, Oregon State University, Corvallis, OR USA

**Keywords:** Microbiome, Bacterial, Diversity, Soybean, Nodule, *Bradyrhizobium*, *Pseudomonas*, Water, Cultivar

## Abstract

**Background:**

Soybean (*Glycine max*) and other legumes are key crops grown around the world, providing protein and nutrients to a growing population, in a way that is more sustainable than most other cropping systems. Diazotrophs inhabiting root nodules provide soybean with nitrogen required for growth. Despite the knowledge of culturable *Bradyrhizobium* spp. and how they can differ across cultivars, less is known about the overall bacterial community (bacteriome) diversity within nodules, in situ. This variability could have large functional ramifications for the long-standing scientific dogma related to the plant-bacteriome interaction. Water availability also impacts soybean, in part, as a result of water-deficit sensitive nodule diazotrophs. There is a dearth of information on the effects of cultivar and water status on in situ rhizobia and non-rhizobia populations of nodule microbiomes. Therefore, soybean nodule microbiomes, using 16S rRNA and *nifH* genes, were sampled from nine cultivars treated with different field water regimes. It was hypothesized that the nodule bacteriome, composition, and function among rhizobia and non-rhizobia would differ in response to cultivar and soil water status.

**Results:**

16S rRNA and *nifH* showed dominance by *Bradyrhizobiaceae*, but a large diversity was observed across phylogenetic groups with < 1% and up to 45% relative abundance in cultivars. Other groups primarily included *Pseudomonadaceae* and *Enterobacteriaceae*. Thus, nodule bacteriomes were not only dominated by rhizobia, but also described by high variability and partly dependent on cultivar and water status. Consequently, the function of the nodule bacteriomes differed, especially due to cultivar. Amino acid profiling within nodules, for example, described functional changes due to both cultivar and water status.

**Conclusions:**

Overall, these results reveal previously undescribed richness and functional changes in *Bradyrhizobiaceae* and non-rhizobia within the soybean nodule microbiome. Though the exact role of these atypical bacteria and relative variations in *Bradyrhizobium* spp. is not clear, there is potential for exploitation of these novel findings of microbiome diversity and function. This diversity needs consideration as part of bacterial-inclusive breeding of soybean to improve traits, such as yield and seed quality, and environmental resilience.

**Electronic supplementary material:**

The online version of this article (10.1186/s40168-019-0676-8) contains supplementary material, which is available to authorized users.

## Background

There are typically increases in plant health, yield, and resilience when an effective association is established between soybean and beneficial nodule bacteria [1, 2]. Especially well known are the positive effects of *Bradyrhizobium* spp. on soybean nodule formation and plant tissue incorporation of bacterial-fixed nitrogen [3]. Different species and strains of *Bradyrhizobium*, however, have different effects on the growth and yield of soybean [4, 5]. Important differences in the effects of various biovars and strains of individual *Bradyrhizobium* spp. on soybean cultivar growth and drought tolerance have been established [6–8]; however, less is known about the natural community variability in nodules that occur when soybean is grown under typical conditions in soils of agroecosystems. Describing and understanding the variability of plant-diazotroph interactions in soybean is the first step to deciphering mechanisms of the symbiosis that eventually lead to the breeding of elite soybean and managing nodule bacteriomes.

Despite evolving as a unique ecological niche for nitrogen-fixation through hosting a symbiotic relationship between the host legume and diazotrophs, a question remains as to the diversity of nodule diazotrophs and whether they share nodules with other genera and families of bacteria. The traditional view of legume-bacteria symbiosis stems from formation and colonization of root nodules by diazotrophs [9]. Culture-dependent sampling and microbiome sequencing, however, have also described the occurrence of atypical bacterial taxa, biovars, and strains that may inhabit nodules of soybean and other populations of legumes [10–12]. These include representatives from across phyla/classes/families, such as *Firmicutes* and *Gammaproteobacteria*. A major gap remains concerning the extent of the presence of these atypical nodule inhabitants using non-cultivable methods, the variation in these communities across soybean cultivars, the functional roles they may play, and the plant-bacterial response to abiotic environmental stressors.

Related to environmental stressors, drought resilience in soybean has naturally been linked to bacterial driven nitrogen fixation. Nitrogen fixation itself is considered the so-called canary in the coal mine, or a sensitive indicator that precedes declining soybean growth in response to drought or other environmental stressors [13–15]. Drought-tolerant nitrogen fixation, moreover, has been predicted to be the trait most pertinent to soybean yield [16]. In addition, recent evidence is suggesting that root nodule communities may not be limited to diazotrophs [10]. Determining the type of bacteriomes—typical diazotrophs and atypical bacteria—that inhabit soybean root nodules, and how they are impacted by changing cultivar and soil water status is a foundational step in linking soybean nodule bacteria with function and physiology that ultimately feedback to effect soybean crop resilience.

Building on decades of research studies describing the interactions between strains and populations of *Rhizobiaceae* species [17], the first objective of this research was to describe the variability of the soybean-nodule bacteriome structure across nine diverse cultivars and responses to changing water status. Because of their potential functional role, there is a need to fill this major research gap in knowledge about the full suite of bacteria (non-cultivable) that reside in nodules. Thus, the main hypothesis for this study was that nodule bacteriome structure, composition, and function would differ in response to cultivar and soil water status. Consequently, the second objective was to describe whether non-*Bradyrhizobium* (non-rhizobia) bacteria are found within nodules and how they vary structurally and functionally across cultivars and water regimes. In this study, we performed nodule microbiome analyses of the overall bacterial communities using the 16S rRNA gene, in addition to diazotroph populations using the *nifH* iron-nitrogenase gene. We also extracted nodule amino acids and conducted metabolic functional prediction to link the changes of nodule microbiome structure to biological changes in the plant-bacterial interaction.

## Results

### Sequencing summary

A customized QIIME pipeline was utilized to determine bacterial diversity across soybean rood nodule samples. In the 16S rRNA dataset, 7.23 million sequences were obtained at a quality threshold of 30, having an average merged length of 385 base pairs (bp) after removing adapters and primers. Following removal of chloroplast and mitochondria-related sequences, 3.88 million sequences remained, with an average 71,799 sequences per sample. These reads were clustered into 4068 OTUs. For subsequent analyses, a threshold of 5200 sequences per sample was used. NIR17 and NIR25 were originally lowly sequenced, and each had only 32 and 136 reads following quality control, so they were excluded from further analyses.

Following quality control preprocessing steps, the *nifH* dataset had 1.58 million high quality reads with a mean length of 318 bp. The average number of reads per sample was 25,400. The reads were clustered into 3257 OTUs at a threshold of 0.97. After applying a threshold of 4295 sequences per sample, NIR1 and NIR20 were excluded from downstream analyses as they had only 198 and 94 reads, respectively.

### 16S rRNA bacterial communities alpha diversity

**Fig. 1:**
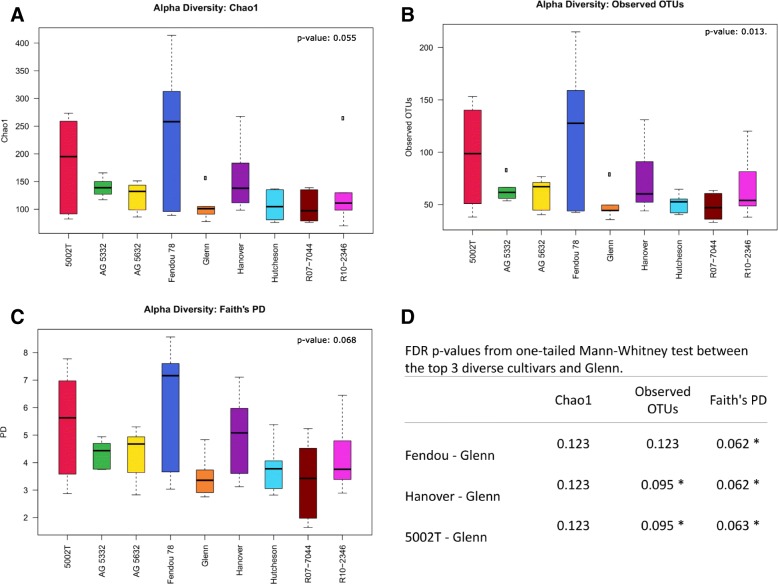
Boxplots showing the differences of alpha diversity indices between cultivars. Three alpha diversities indices were used, **a** chao1 and **b** observed species show the species richness, while **c** Faith’s phylogenetic diversity includes the evolutionary relationship component. *p* values shown were determined using MRPP. Pairwise significant differences as tested by one-tailed Mann-Whitney FDR (*p* value < 0.1 highlighted with an asterisk) between the top 3 diverse cultivars and the least diverse are given in (**d**)

When grouped by cultivar, the diversity indices revealed higher levels of diversity among samples from Fendou-78, 5002T, and Hanover, respectively, when compared to the lower bacterial diversity cultivars (Fig. 1**)**. The remaining cultivars had similar indices of richness and diversity. The sampling was deemed to have sufficient depth, supported by an average Good’s coverage estimation of 99.8%. Non-parametric comparison using MRPP of the Euclidean distances of alpha diversity indices revealed a significant effect of cultivar (*p* value < 0.1) [18]. There were, however, higher levels of nodule community variability, especially in Fendou-78, 5002T, and Hanover, which suggests stochastic biological interactions between soybean and nodule bacteria in this large sample of taxa soybean nodules.

### Taxonomy summary

**Fig. 2:**
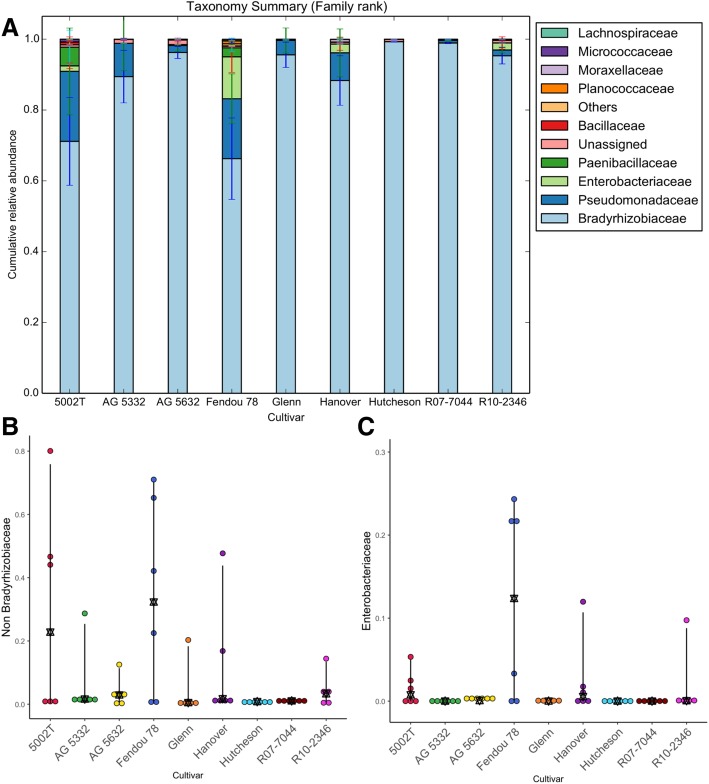
Taxonomic summary of cumulative relative abundance of nodule bacterial communities of the nine plant cultivars. Taxonomies are shown at the family rank where **a** shows the top 10 most abundant families. In a further break down of this diversity, **b** shows the relative abundance of non-*Bradyrhizobiaceae* (atypical diazotrophs), and **c** shows *Enterobacteriaceae* that was significantly affected by cultivar (Kruskal-Wallis, *p* value = 0.03)

Relative abundances at the family taxonomy level revealed novel results regarding the widespread presence of atypical nodule bacteria. Family taxa varied where averages of *Bradyrhizobiaceae* contributed as little as 66% in Fendou-78 but as much as 99% in Hutcheson (Fig. 2). *Pseudomonadaceae* ranged from being nearly undetected in Hutcheson to 19.8% in 5002T. *Enterobacteriaceae* were mainly present in Fendou-78 (11.8%), Hanover (2.5%), R10-2346 (2.0%), 5002T (1.6%), and nearly absent in the other five cultivars (Fig. 2c). *Paenibacillaceae* were most prevalent in 5002T (5.3%) and Fendou-78 (2.5%). Aside from *Bradyrhizobiaceae,* all other families had a high coefficient of variation. *Pseudomonadaceae* and *Enterobacteriaceae* had a 113% and 180% coefficient of variation across treatments, respectively, compared to just 13.6% for *Bradyrhizobiaceae.* There was a further wide range of interesting changes in the abundances of these taxa within the cultivars when the effect of irrigation is considered. The abundances of non-*Bradyrhizobiaceae* reach up to 40% in some treatments. A summary of these changes has been given in (Additional file 1: Figure S1). There was thus a diversity of family-level bacteriomes albeit with high variation across cultivars.

### Diazotrophic populations (nifH) alpha diversity

The *nifH* gene was used as a marker to survey the nitrogen-fixing communities residing within the nodules. Alpha diversities were calculated using chao1 and observed species indices. The rarefaction plots showed that the non-irrigated samples had a higher alpha diversity than the irrigated samples (Additional file 1: Figure S2). This was also reflected in the non-parametric *t* tests, which showed a significant difference due to irrigation for chao1 and observed species indices (*p* < 0.01). Unexpectedly, there were no differences in the diversity and richness of diazotrophic populations when assessed by cultivar or the interaction of cultivars and the irrigation treatment.

### Taxonomy summary

The diazotrophic populations were overwhelmingly dominated by *Proteobacteria*, representing 99.8% of OTUs. *Firmicutes* accounted for the remainder minority of the phyla. Of the *Proteobacteria* sequences, more than 99% were represented by *Bradyrhizobium*. *Bradyrhizobium* was split primarily between two taxa, *B. japonicum* and *B. elkanii*, accounting for 63.2% and 34.5%, respectively, whereas the other 2% of OTU clustered into other unidentified *Bradyrhizobium* genera (*n* = 52). When taking the irrigation treatment into consideration, all cultivars have shown mixed changes. For instance, in 5002T, there was little change in *Bradyrhizobium* species relative abundances due to irrigation; *B. japonicum*, for instance, remained fairly stable from 63.5% to 62.3% between the non-irrigated to irrigated treatment (*p* value = 0.35). Fendou-78 exhibited a large change where *B. japonicum* decreased from 71.4% to 1.1% and *B. elkanii* increased from 26.6% to 91.7%, in the non-irrigated compared to irrigated samples, respectively (*p* value = 0.1). Most of the other cultivars showed a similar change in direction but the magnitude of change was smaller. On the other hand, AG-5632 was described by the inverse of the above result. It had an increase in *B. japonicum* from 52.2% in non-irrigated compared to 96.2 % in the irrigated treatment, whereas *B. elkanii*-related taxa decreased from 44.9% to 3.3% between non-irrigated to the irrigated samples (*p* value = 0.05). As expected, these results show that diazotrophs show specific cultivar effects and interactions with cultivar that are sensitive to water status.

**Fig. 3:**
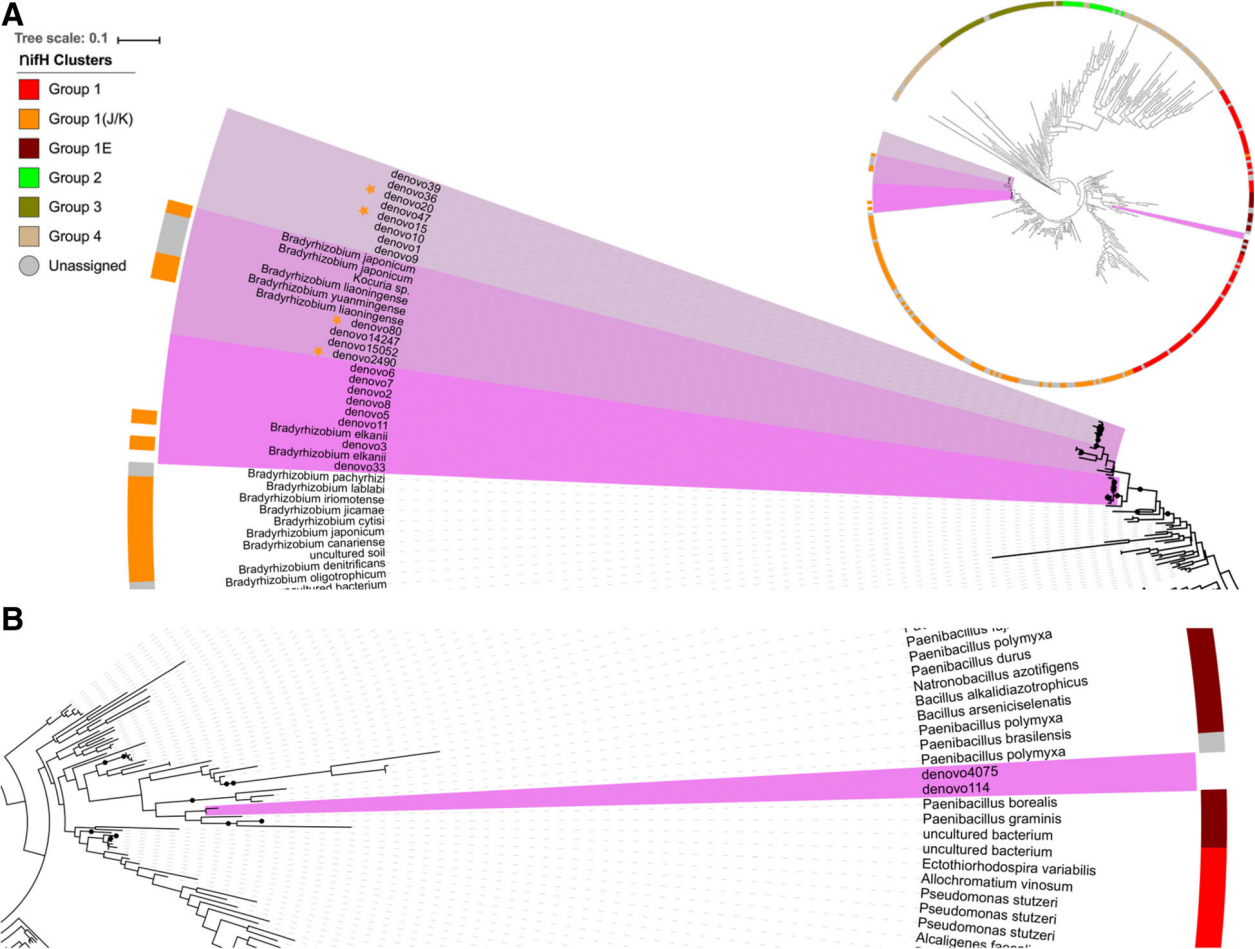
Phylogenetic tree showing the evolutionary diversity of the sequenced nifH genes. An overall view is given of a representative set of all nifH genes. Panel **a** shows the placement of some of the most abundant *Bradyrhizobium*-related nifH genes. Different shades of pink highlight the distinct subclades of each species/strains present in the study. **b** Genes closely related to *Paenibacillus* are also found in the nodules but in rare abundances. Black nodes on branches denote bootstrap confidence values above 60%. The colored strips outside the tree leaves show the *nifH* cluster assignment according to (Heller et. al) naming convention, where only subgroups 1E, 1J/K distinguished in group 1. Significantly changing OTUs in response to irrigation are highlighted with stars (FDR *p* value < 0.05)

A Kruskal-Wallis test performed on OTUs revealed nine OTUs with significant variation in abundance due to irrigation. Four of these OTUs were among the most abundant OTUs that made up 95% of the total sequenced reads (Fig. 3). These OTUs belonged to either *B. japonicum* or were unclassified *Bradyrhizobium*. No OTUs were revealed to have relative differences in OTU abundance due to cultivar only. These results indicate that the most abundant diazotrophs and their interactions with plants have different sensitivities to water status.

### Phylogenetic diversity

To assess the evolutionary diversity of the diazotrophs within the current study’s samples and their relationship to well-known diazotrophs, a phylogenetic tree was constructed. OTUs comprising about 95% of the total population in addition to the top OTUs from *Firmicutes* were used to create a phylogenetic tree that encompasses a representative member of the major *nifH* groups (Fig. 3) [19]. All of the *Bradyrhizobium* OTUs classified in this study formed a monophyletic clade with the 1J/K group based on the *nifH* gene, which includes most types of *Bradyrhizobium*. Within that group, they further form three separate sub-clades. The first clade does not contain other types of identified *Bradyrhizobium.* This indicates the potential existence of novel species or strains of *Bradyrhizobium.* The second clade clustered with the *Bradyrhizobium japonicum* group, while the third clade grouped with *Bradyrhizobium elkanii*. The two OTUs belonging to *Firmicutes* were placed with *nifH* group 1E, where they are closely related to *Paenibacillus.* Given the negligible abundance of *nifH* genes in these latter taxa, they likely did not contribute significantly to the nitrogen fixating population of the bacteriome or the process of nitrogen fixation.

### Community beta diversity

**Fig. 4:**
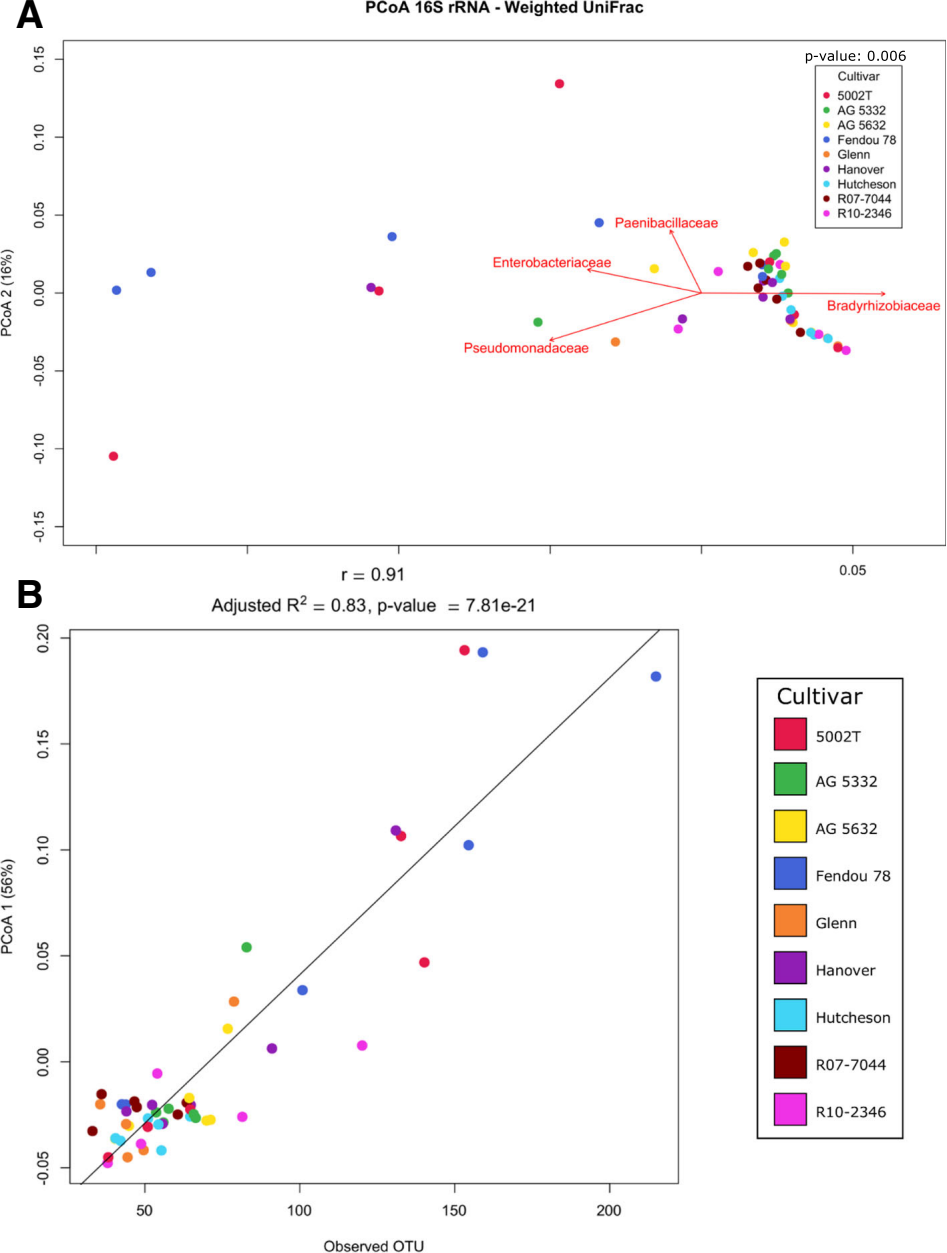
Visualization of the beta-diversity showing the differences between cultivars. **a** PCoA of the beta-diversity between different cultivars based on the UniFrac distances of the nodule bacteriome of as determined by the 16S rRNA gene. Biplot shows the correlation of major family-level taxa with the samples. Statistical significance of the cultivar effect using MRPP is shown. **b** Scatter plot showing a direct positive correlation between the first principal coordinate and the observed species diversity index

**Fig. 5:**
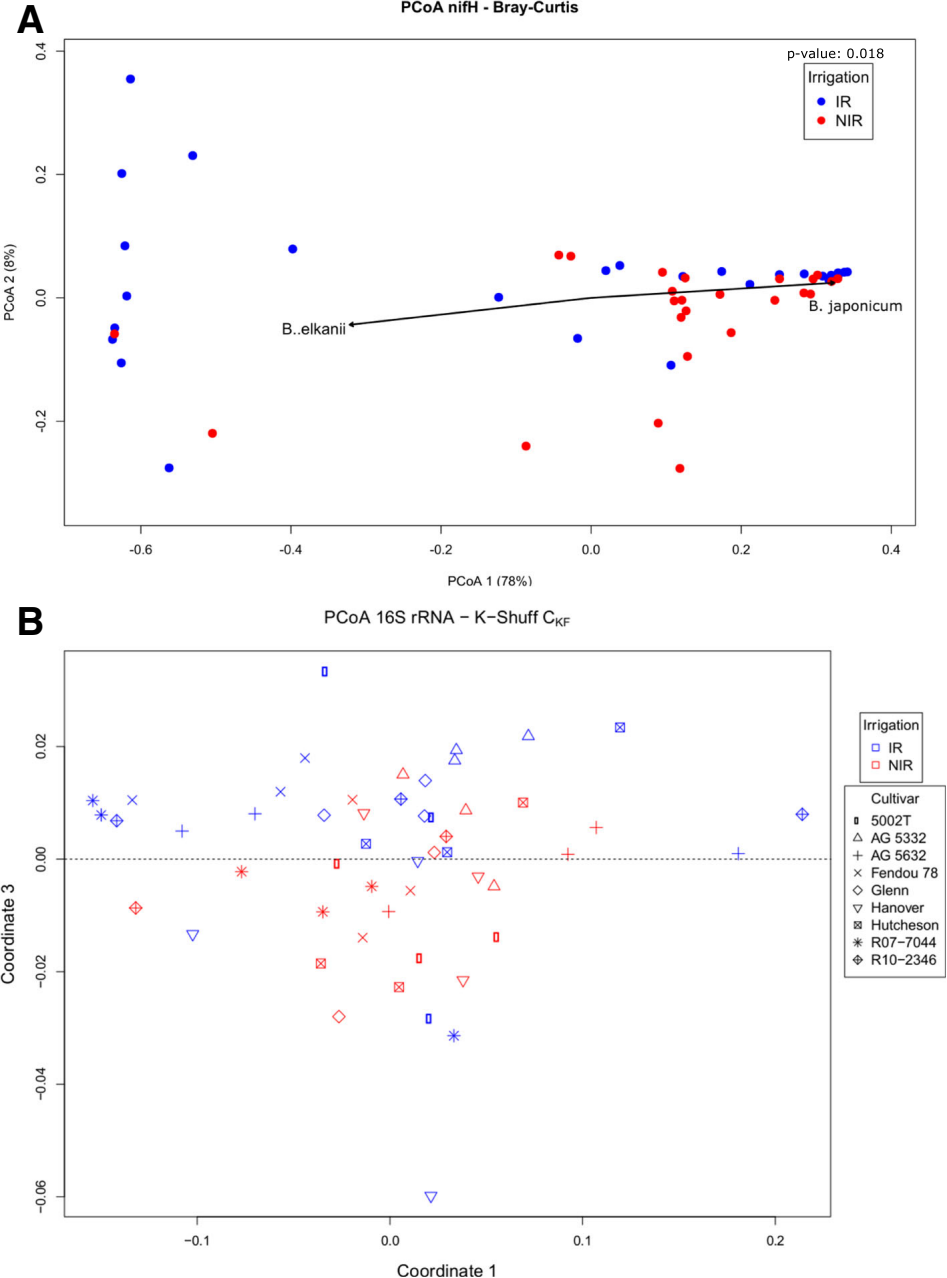
The effect of irrigation on the microbial communities’ structure and composition. **a** PCoA of Bray-Curtis distances for the nifH-based diazotrophs, showing the beta-diversity changes between irrigation treatments. Biplot shows the correlation of the two major *Bradyrhizobium* species with the samples. Statistical significance of the irrigation effect using MRPP is shown. **b** PCoA of the cross K-functions (_C_K_F_) derived from K-Shuff for the 16S rRNA genes. The plot highlights a separation between the samples based on the irrigation treatment, as delineated by the dashed line. K-Shuff statistical testing revealed a significant effect for all experimental factors (*p* value = 0.001)

Taxonomic summaries of the 16S rRNA-based bacteriome revealed variation in bacterial taxa due to cultivar, and this was supported by multivariate statistical tests that were based on the weighted UniFrac distances (Fig. 4; Table 1) and water status, as shown by K-shuff (Fig. 5b). K-shuff is based on a distance measure, which is set as the evolutionary distance between gene sequences, and is thus sensitive to differences between samples [20]. Both MRPP and ADONIS showed a significant difference between cultivars, but not for water status nor the interaction of water and cultivar. To identify the effect of each factor on the bacteriome in the nodules, weighted UniFrac distances of the evenly rarefied OTUs across the samples were displayed in the ordination space using principal coordinates. The first principal coordinate accounted for 56% of the variability, and samples from two cultivars were distinct along that axis (Fig. 4a). Though no clear separation was observed due to water status using UniFrac (Fig. 4a; Additional file 1: Figure S1), K-shuff distinguished these effects (Fig. 5b). It is thus concluded, based on evolutionary distances, that water and cultivar both impacted community beta diversity (community structure).

**Table 1:** Significance of treatments on samples beta diversities and amino acid profile as determined by ADONIS and MRPP

	Test	Irrigation	Cultivar	Irrigation × cultivar
nifH (Bray-Curtis)	ADONIS	**0.038**	0.358	**0.047**
	MRPP	**0.018**	0.487	**0.046**
16S (Weighted Unifrac)	ADONIS	0.985	**0.008**	0.146
	MRPP	0.693	**0.006**	0.069
Amino acid profile	ADONIS	**0.007**	**0.014**	0.089
	MRPP	**0.001**	0.113	**0.007**

Samples are grouped based on the effects of irrigation, cultivar, and their interaction. Bold values indicate *p* value < 0.05. *p* values were computed using 999 permutations

There were two cultivars that were most separated from the rest, Fendou-78 and 5002T (Fig. 4a, b). To statistically confirm the contribution of each cultivar to the significance of the effect, multivariate analysis of the homogeneity of cultivar variances were performed using PERMDISP. 5002T, Fendou-78, and Hanover had higher beta dispersion (departure from average) values than other cultivars (Additional file 1: Figure S3). Follow-up analysis using Tukey’s honestly significant difference (HSD) for the pairwise comparisons between the cultivar nodule bacteriome revealed 5002T to be the cultivar most different from other cultivars, followed by Fendou-78 and Hanover. This shows that these three cultivars stand out from the others due to their community composition, albeit the within-cultivar variability was high.

Multivariate statistical analyses of the diazotrophic populations, based on *nifH* gene abundance profiles using MRPP and ADONIS, showed significant effects of both water status and the interaction between water status and cultivar type (*p* value < 0.05) but not for cultivar type alone. Visualization of the principal coordinates of the Bray-Curtis distances based on the *nifH* OTUs revealed a clear separation between samples based on irrigation treatment along the first coordinate, which accounted for 78% of the variation (Fig. 5a). This shows that these nitrogen-fixing populations where more sensitive to water status than the overall bacteriome, confirming an expectation of this study, that diazotroph nodule communities are sensitive to soil and plant water status.

### Functional analyses

Nodule bacteria are known for their nitrogen-fixing abilities in legumes like soybean. However, in the presence of a high abundance of non-nitrogen-fixing bacteria, there could be some functional and metabolic differences. To determine the functional potential of nodule communities and possible contributions to the plant or plant-bacterial interactions, PICRUSt was used to describe the functional profile of the nodule bacteriome, which was followed by further statistical analysis and visualization in STAMP. Based on KEGG classification, after excluding human disease and other eukaryotic-related system pathways, 28 level 2 functional pathways and 118 level 3 pathways were found to be statistically significant due to cultivar (Additional file 1: Table S1). Nitrogen related pathways, in particular, like amino acid metabolism, metabolism of other amino acids, and nucleotide metabolism were found to be significantly different in nodules due to cultivar. In level 3 pathways, “Phenylalanine, tyrosine and tryptophan biosynthesis”; “Valine, leucine and isoleucine metabolism”; and “D-Arginine and D-Ornithine metabolism” in addition to “Cysteine and Methionine metabolism” were found to be significantly different between cultivars. In addition, “sucrose and starch metabolism” was more enriched in Fendou-78 compared to the nodules of other cultivars. No pathways were found to be significantly different between the irrigated and non-irrigated samples on any KEGG level. This may seem contradictory to the differences in diazotroph populations associated with water status; however, this may be due to a lack of sensitivity of PICRUSt to differences in function at the species level. Overall, these results indicate that based on the bacteriome, there are changes in amino acid and carbon cycling potential that would be expected to differ due to cultivars with different nodule bacteriomes.

**Fig. 6:**
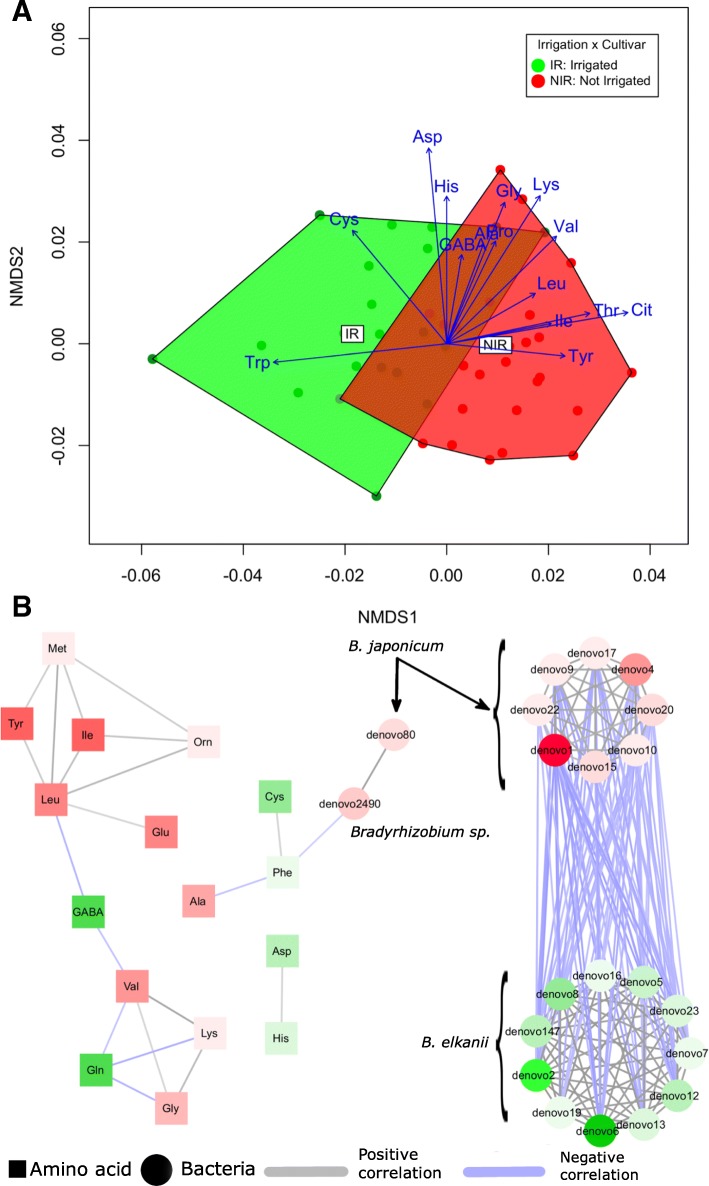
The functional consequences of the irrigation treatment on the amino of the irrigation treatments on the amino acids. **a** shows the irrigation effect on the amino acids profile through an NMDS. Individual samples are resembled with filled circles. Amino acid vectors were overlaid, where only significantly correlated amino acids (*p* < 0.05) shown. Vectors pointing to the left direction along the first axis are enriched in the irrigation samples, whereas vectors pointing to the right side are enriched in the non-irrigated samples. **b** Integrates the amino acid profile and nifH OTU cluster abundances. The figure shows both the bacteria (circles) and the amino acid (square) where the color would be green if they are enriched in the irrigated treatment or red when enriched in the non-irrigated treatment. Gray links indicate a positive co-occurrence whereas a purple link indicates a negative co-occurrence

An amino acid profiling of nodules from all plants was also conducted. The amino acid profile not only showed a significant irrigation effect (Fig. 6), but also a strong interaction between both cultivar and irrigation amino acid profiles (Table 1). Though somewhat different than PICRUSt, the former focused on differences in predicted gene abundances and thus potential functional changes, whereas amino acid profiling was a direct description of in vivo functional change. Overall, 15 amino acids were strongly related to the community variation (*p* < 0.05) (Additional file 1: Table S2). Tryptophan and aspartic acid had the highest correlations and were more enriched in the irrigated samples whereas threonine, tyrosine, and non-proteinogenic citrulline were enriched in the non-irrigated samples. Although these results highlighted a stronger irrigation effect than PICRUSt, they have also validated the functional prediction of PICRUSt based on cultivar variation interacting with the effect of water status, wherein the latter case strong differences between *Bradyrhizobium* spp. were detected.

### Integrated network analysis

In order to have a more in-depth view on the associations between the different types of bacteria and the amino acids, a co-occurrence network, analysis based on the irrigation treatment, was conducted combining all datasets. Following filtering criteria, the network resulted in 37 nodes, 21 for nifH OTUs and 16 for amino acids. No 16S rRNA taxa were included due to lack of significant correlations under irrigation. The network nodes were organized into four modules (Fig. 6b). Most nifH OTUs clustered in one module with a high clustering coefficient of 0.956 and a high degree of connectivity. This is due to the strong and direct collective effect of irrigation on diazotrophs. The module was further divided into two sub-modules based almost exclusively on the species. This further supports the beta-diversity analysis (Fig. 5a). Whereas, the amino acids clustered into two modules with a 0.572 clustering coefficient and average number of connections of just 3.1. This suggests the functional specialization of the different amino acids. However, just one module combined the amino acids and the nifH clusters together, and it contained three amino acids and two diazotrophs. This is indicative of a lack of a significant association between the diazotroph type and the amino acids. This could be the result of some similar functional changes between the two species.

## Discussion

The nodule microbiome of soybean roots from nine diverse cultivars was studied using high-throughput sequencing. As hypothesized, a unique finding was that the structural diversity of *Bradyrhizobium spp.* and many non-*Bradyrhizobium* bacteria (e.g., *Pseudomonas* and *Enterobacteriaceae*) differed with cultivar. The extent of variation across cultivars was greater than predicted; however, where in some cases, nodules were composed of ~ 40% atypical non-*Bradyrhizobium* spp. This degree of variation generally and across several cultivars have not been previously reported and point out that there is considerable research needed to better understand the role of these bacteria, currently considered to be atypical, in the plant-bacterial interaction. Water status, as hypothesized, also had an impact on the overall bacteriome structure, and as expected, the diazotroph nodule population changed, supporting the overall notion that *Bradyrhizobium* spp*.* are sensitive to water status [21–23]. It cannot be ruled out, however, that the change in the *Bradyrhizobium* spp. was wholly or in part a result of the plant response to water deficit. The most novel and perhaps most important finding was the high bacterial family-level diversity in nodules that differ based on cultivar and water status (Figs. 4 and 5). As hypothesized, these changes in bacterial diversity due to cultivar and water were also described by indicators of functional change. This would be expected to have major biological and ecological consequences for understanding soybean-bacterial interactions and traits related to the soybean-microbiome, for example, such as seed yield and nitrogen fixation capacity.

### *Bradyrhizobium* population variation in nodules of soybean

The variable occurrence of two dominant 16S rRNA based OTUs closely related to *Bradyrhizobium* spp., several subdominants (1–2%), and a wide variety of rare OTUs across nine different cultivars suggest unique ways of interaction that occur between plant-bacterial mutualists. The observation of these *Bradyrhizobiaceae*-related OTUs describes a novel observation of very high diversity and brings to light new questions about the roles played by these rare taxa. These results build on the culture-based findings of 543 nodules from 19 farms in Wisconsin that described 35 distinct strains belonging to 7 serogroups [24] but beg the question of how bacterial diversity may alter soybean traits. Plant species richness is considered to bring resilience and ecosystem stability related to function [25], but whether high diversity in nodules provides a diversity of bacterial genes that benefit plant resilience under different environmental conditions remains an important but open question. The changes in communities with water status might result in substantial changes in genetic composition and ultimately bacterial functioning. Well-controlled experiments, however, will be needed to test this hypothesis.

The discussion above stresses the importance of the plant-bacterial interactions, but distributions of *Bradyrhizobium* spp. cultured from soil have been shown to be correlated with latitude. In the north, *B. japonicum* serogroups overwhelmingly dominate while those of *B. elkanii* tend to dominate in Southern states (e.g., Alabama) where *B. elkanii* composed up to 90% of total *Bradyrhizobium* populations [26]. Similar latitudinal patterns were observed in Japan [27]. These studies suggest that biogeography and climatic factors such as temperature play a role in *Bradyrhizobium* spp. distribution in soil. The current study (Virginia) whereby *B. elkanii* tends to dominate relative to *B. japonicum* resemble those of the adjacent state of North Carolina (58.3% to 41.7%, respectively) (Additional file 1: Figure S4) and suggest that soils may also play a role in providing the source of bacterial taxa. However, it is unclear if soil differences reflect soil properties or inherent legacy effects of past cultivars of soybean that are best suited to a region. As shown herein and elsewhere, cultivars themselves clearly exert an influence [28] and help to explain variation with latitude. Thus, regional bacterial patterns are partially the result of the type of cultivar grown in a region, along with the effect of the environment (soil, temperature).

### Non-*Bradyrhizobium* bacteria in soybean nodules

The most prominent result of this study was the relatively high abundance of what would be traditionally atypical non-nitrogen-fixing bacteria obtained from nodules of at least two soybean cultivars. Though some bacterial strains belonging to *Pseudomonadaceae* and *Enterobacteriaceae* are known to be equipped with genetic machinery required for nitrogen fixation [29, 30], the divergent *nifH* paralogs of these bacteria evolved to function in a different ecological context with different plants. In this study, *nifH* results do not support the idea that these atypical bacteria are nitrogen-fixing variants since more than 99.5% of the *nifH* genes belonged to taxa closely related to *Bradyrhizobium* spp. (Fig. 3). Though horizontal gene transfer has been reported to occur between *Bradyrhizobium* and *Bacilli* [31], there is generally little information in the literature on this phenomenon. It thus, currently, seems that non-rhizobia taxa in this study are unlikely to possess *nifH* genes from *Bradyrhizobium* or other sources. The functional impact of these non-rhizobia bacteria is not known but could have important impacts on the energetics, symbiosis, and nutrient exchange for soybean.

Non-*Bradyrhizobium* bacteria have been reportedly isolated from nodules of soybean, but their high abundance in some cultivars in this study raises questions about their role in nodule function. There is sparse data in this regard, but microbiome sequencing of nodule bacteria from a soybean and an alfalfa plant previously showed a range of non-rhizobia of 1–20% [10, 11]. It should be noted that in these studies, the experiments were under more controlled conditions where they were planted in a greenhouse or inoculated. In this study, we report more than 40% relative abundance of non-*Bradyrhizobium* bacteria in one treatment (Additional file 1: Figure S1). In addition, this diversity was isolated from pooled plants that incorporate nodules without differentiating their lifetime stage. Nonetheless, this novel high diversity and occurrence of these taxa seemed to show a very low correlation with yield (Additional file 1: Figures S5 and S6), and the net outcome of their presence might be neutral; however, this supposition is based on a temporally small scale and so the potential role of these taxa can only really be supported with controlled studies.

*Serratia* sp. (*Enterobacteriaceae*) has previously been shown to improve soybean nodulation by *Bradyrhizobium*, and there are mixed reports regarding the nodulation effects of *Pseudomonads* [32, 33], though there is evidence of their promotion of *Bradyrhizobium* growth through the production of Fe-chelates [34]. The variation across cultivars cannot discern the cultivar-bacterial interaction. Nonetheless, beneficial roles of related bacteria to those observed herein have been shown in the rhizosphere [35]. It is perhaps their high abundance in the rhizosphere that may catalyze their movement into nodules. *Pseudomonas* and *Enterobacteria* in the rhizosphere have been shown to solubilize organic phosphorus into phosphate and support its availability to plants [36, 37]. They also have a strong role shaping nodulation competition between different *Bradyrhizobium* strains under low-iron availability, thus affecting the soybean-diazotroph-soil biogeochemical interactions [38]. Rhizosphere *Bacilli* have also been shown to improve the biomass of soybean plants when they were co-inoculated with *Bradyrhizobium*, yet they were not shown to confer the same advantage when inoculated alone [39]. *Paenibacilli*, furthermore, were shown to reduce water-deficit stress in other legumes by decreasing stress-signaling hormones [40]. While these bacteria have plant growth promoting traits in the rhizosphere, their roles inside the nodules need further investigation.

Differences in the bacteriome due to water status were supported by K-Shuff analysis (Fig. 5b, Additional file 1: Figures S1). The shifting role of the mixture of typical and atypical nodule bacteria may also be mixed, but their variation across water treatments suggests that further studies into this dynamic and variable system related to water stress, or possibly other unaccounted for environmental variables, are needed. Another question is why such a large variation (i.e., 1–40%) in nodule inhabitation by non-rhizobia taxa was observed due to the effects of cultivar and water (Additional file 1: Figures S1). While the idea of symbionts that exist as free-riders in the legume-rhizobium interactions is known, soybean also has its own molecular means to select for bacteria and penalize uncooperative nodules [41–43]. Yield and diversity across cultivars did not seem to be related to the occurrence of these taxa, though there were some weak to moderate correlations between yield and the most abundant OTUs (Additional file 1: Figures S5 and S6). Given the complexity of the nodule communities with many diverse taxa, there may be multiple positive and negative effects occurring concurrently and change with cultivar [44]. Studies in other legumes such as *Lotus japonicus*, the model plant for the study of legume-rhizobia communication, suggested that plant gene expression may actually facilitate the inhabitation of these non-nitrogen-fixing nodule endophytes [45]. While the role of these non-rhizobia bacteria is still not understood for soybean, their occurrence in the nodules may be due to genetic traits of the cultivars or *Bradyrhizobium*. Changes in nodule bacteriome due to water status may be part of a plant-bacterial response mechanism to aid mutual acclimation to new environmental conditions [46–48]. Approaches such as genome-wide association, however, would be needed to account for the biological significance of these atypical taxa could provide gene targets for breeding of soybean for water stress, cultivar, and other soybean-microbiome traits.

### Nodule bacteria functional changes

Functional profiling of root nodule amino acid composition, as hypothesized, was shown to be associated with cultivar and water status. First, amino acids are key constituents of nitrogen exchange between nodule bacteroid and soybean, and they can thus provide information on functional changes between the mutualistic partners. Excess nitrogen, for example, in the form of amino acids can be exported from the leaves and translocated to feedback and reduce nodule-fixing activity [49]. Amino acid changes cannot directly support the idea of translocation noted above (Fig. 6), but they do indicate a broad pattern of functional change indicative of the water status and the interaction of cultivar with water. Tryptophan, for example, was enriched with greater soil water, concomitant with less bacterial diversity and greater relative abundances of *Bradyrhizobium* (Additional file 1: Table S2). High levels of *Bradyrhizobium*-synthesized tryptophan in the nodule has a strong role in stabilizing the symbiotic relationship with legumes [50]. Second, functional profile prediction using PICRUSt and STAMP found that several pathways that can modulate soybean-nodule interactions varied significantly across cultivars. Amino acid metabolism and metabolism of other amino acids (e.g., non-proteinogenic) pathways were underrepresented in the microbiomes of Fendou-78 and 5002T compared to other cultivars, whereas the opposite was true for nucleotide metabolism (Additional file 1: Figure S7). They both happen to be the cultivars with the largest nodule size (Additional file 1: Figure S8). It has been observed that larger nodules and perhaps similarly larger bacterial communities per nodule may support acclimation to changing environmental conditions [51], supported herein furthermore by possible mechanisms of metabolic and functional acclimation (e.g., amino acid change) in nodules during water deficit.

The pattern of amino acid change observed herein provides independent confirmation of concurrent bacteriome and nodule metabolic changes resulting from cultivar and water effects. Although the integrated network analysis supports the strong effect of irrigation on the diazotrophs (i.e., *Bradyrhizobia*), it does not support their direct effect on the majority of amino acids. On the other hand, cultivar variations were previously shown to reflect the active role of the bacteria-mediated nodule metabolism through amino acid cycling [52]. Based on in vivo amino acids profiling and PICRUSt, functional changes in nodule communities are associated with both cultivar and water status (Fig. 6a, Table 1; Additional file 1: Table S1). These results mirror a corresponding outcome of the human microbiome study, where large individual variations in bacteriome structure existed across sampled individuals, whereas the corresponding functional variations reflected differences such as environment and host genetics [53]. This is an important and rare ecological field-based finding that links community and populations of the bacteriome and nodule function within soybean cultivar and field water status. These results thus have implications for the outcome of soybean growth and resilience in the face of environmental variation.

## Conclusions

In summary, the field-based results here provide a novel view of the diverse nodule bacteriome and the diazotrophic population inside the nodules of several soybean cultivars with a wide assortment of genotypes. The main highlight of this study was that many types of non-rhizobia taxa appear to be relatively highly abundant (*Pseudomonas* and *Enterobacteria*), making up previously unreported large proportions of the nodule community across a few cultivars. It is not known if their presence is related to potential biological and biogeochemical cues resulting from plant-bacteria-soil interactions, however. The impact of water status and cultivar was observed on the broader bacteriome structure, but unsurprisingly, the water-sensitive population structure of *Bradyrhizobium* spp. was much more affected, further enforcing the idea that nitrogen-fixation is sensitive to soil water status, a rare confirmation of this idea that was observed under field conditions. However, the high variance of typical rhizobia and atypical taxa in nodules of cultivars and the sensitive nature of *Bradyrhizobium* to water status indicate that the ecology of soybean-bacterial interactions may be more complex than previously thought. Metabolic and functional level responses, especially those related to amino acid cycling, reflected different variations in broader bacteriome and specialized diazotroph population structures. Whether the non-rhizobia bacteria support, inhibit, or have no effect on soybean growth is a critical question brought about by this research and in major need of further investigation. Based on the field-based findings herein, greater integration of bacterial, phylogenetic, taxonomic, and functional diversity describing soybean-bacterial interactions are in need and, for example, could support both traditional and molecular breeding programs to improve traits, such as yield, seed quality, and environmental resilience.

## Materials and methods

### Experimental design and field setup

The experiment was carried out in Virginia Tech’s Kentland farm (37.1998° N, 80.5644° W), a study site that mimics the environment of a major soybean region (e.g., southeastern coastal plain of the USA). In this study, nine diverse cultivars were studied using a split-plot design with two water status treatments, nine cultivars, and three replicates for a total of 54 samples. Each cultivar-treatment combination was allotted a plot with four rows 30 inches apart and 10 feet long. The outer two rows were used for sampling plants for dry weight measurements and nodule extraction, whereas the inner two rows were reserved for taking measurements and yield calculations. The soybeans were planted in the field in May 2014 and harvested in October 2014. Two watering treatments were applied, non-irrigated and irrigated. For the non-irrigated treatment, the plants were received rainfall. In the irrigated treatment, the plants received rainfall and two applications of ~ 1-inch following episodes of drought in June and July. Information about the growing season climate data has been compiled from the onsite weather station (Additional file 1: Table S3). Bulk soil water content was measured after drying in the oven at 100 °C for 48 h (Additional file 1: Table S4). A summary of the study design and workflow is presented in Additional file 2: Figure S10.

### Soybean nodule cleaning and DNA extraction

For nodule extraction, two plants were harvested from the outer two rows of a plot. To determine the bacteriome, nodules were first extracted from the roots and stored at − 20 °C until DNA extraction. Nodules were sterilized and decontaminated of surface DNA prior to microbial DNA extraction. First, the nodules were rinsed three times in a 0.9% NaCl solution to remove soil particles from the surface where between each rinse, they were rolled on lint-free wipes to remove excess soil. After that, the nodules were soaked twice for 5 min in sodium hypochlorite solution (1.65% *v*/*v*) while mixing by inversion. They were rinsed off in-between the washes. Finally, the nodules were rinsed three times using sterile water. To confirm the sterility, nodules were rolled on yeast-mannitol-agar plates. The plates were incubated at 28 °C for 48 h after which they were inspected and shown to have no colony formation. DNA was extracted from the sterile nodules using the Power Soil DNA isolation kit according to the manufacturer’s guidelines (No. 12888-100, MoBio Laboratories Inc., Carlsbad, CA, USA). Finally, the DNA quality and quantity were evaluated using NanoDrop 2000 spectrophotometer and 0.8% agarose gel electrophoresis. A negative control was used throughout until the PCR reaction.

### DNA sequencing and data analyses

The 16S rRNA libraries were prepared according to Illumina’s 16S Metagenomic Sequencing Library Preparation protocol. Briefly, the 16S rRNA gene was PCR amplified using the forward S-D-Bact-0341-b-S-17 and reverse primer S-D-Bact-0785-a-A-21 [54]. Primer pairs had the Illumina overhang adapter sequences ligated to them (IDT, Coralville, IA, USA). PCR products were cleaned using Agencourt AMPure XP magnetic beads (Cat no. A63881, Beckman Coulter, Brea, CA, USA). A second PCR reaction ligated indexing barcodes for multiplexing the samples from Nextera XT Index Kit v2 set A (Catalog no. FC-131-2001, Illumina Inc., San Diego, CA). PCR products were purified again using the magnetic beads. DNA concentration was determined by fluorometric quantification using the Qubit® 2.0 platform with Qubit dsDNA HS Assay Kit (Life Technologies, Carlsbad, CA, USA). Samples were diluted into equimolar concentrations and sent for sequencing. Sequencing was performed using a 500 cycle v2 kit on the Illumina MiSeq instrument at the Biocomplexity Institute core facility in Virginia Tech (Blacksburg, VA, USA).

The 16S data was preprocessed as described previously [55]. Briefly, demultiplexed reads were quality filtered based on a minimum Phred score of 30 and 100 base pairs length. Barcode adapters and primers were trimmed using cutadapt [56]. Overlapping paired-end reads were merged using pandaseq [57]. Downstream analysis was performed in QIIME v1.8.0 [58]. The representative set of operational taxonomic units (OTUs) were determined using the open-reference OTU picking strategy. Reads were clustered using UCLUST [59] into OTUs at a minimum similarity of 97%. Taxonomic assignments were derived by comparison to the GreenGenes database v13.8 [60]. OTU classifications belonging to mitochondrial and chloroplast 16S rRNA OTUs were filtered preceding this analysis. OTUs significance across different groups was determined using the nonparametric Kruskal-Wallis *H* test implementation in groups_significance.py script using 999 permutations.

For alpha diversity and generation of rarefaction curves, OTU tables were sub-sampled at different depths up to 5200 reads per sample. This led to the exclusion of samples with low numbers of sequences (< 10). The following alpha diversity metrics were used through QIIME interface: PD_whole_tree, chao1, observed species, ACE, Good’s coverage, and Shannon and Simpson indices. Alpha diversities were compared for all groups using MRPP in PC-ORD 6. OTU tables were rarefied at even sampling depth of 5200 for all samples for subsequent analyses. Beta-diversity computations were based on the weighted and unweighted UniFrac distances matrices [61]. The distances were based on the OTU abundance tables evenly rarefied at 5200 sequences per sample and a phylogenetic tree. This required the alignment of the representative set of OTUs using PyNAST [62] and the generation of the tree using FastTree 2 [63]. To test for significant differences between groups belonging to irrigation treatment, cultivar, and their interaction, multivariate hypothesis testing was performed using ADONIS and MRPP with 999 permutations each. A further multivariate analysis of homogeneity of the groups’ dispersions was done using betadisper function from the package vegan and in R [64]. Post hoc analysis was conducted using Tukey’s HSD. All non-parametric tests had 999 permutations. Structural and compositional differences between different libraries were compared using K-shuff [20]. This required preprocessing the reads in mothur [65]. Reads were aligned against Silva database v.123, followed by filtering the alignment, sub-sampling 200 reads per library, and generating a square distance matrix using the dist.seqs function in mothur.

Functional potential of the bacteriome due to cultivar and water status was predicted using PICRUSt v1.0.0 [66]. PICRUST estimates the functional potential based on the normalized 16S rRNA copy numbers derived from the GreenGenes database, hence, all de novo OTUs were removed prior to analysis. Tables were categorized and generated based on level 3 of functional pathway classification at the pathway level in the KEGG database [67]. PICRUST tables were visualized and tested for statistical significance using STAMP v 2.1.3 [68]. *p* values in STAMP were corrected using the Benjamini-Hochberg procedure.

The *nifH* library was prepared similarly to the 16S rRNA albeit with the following changes. PolF (5′-TGCGAYCCSAARGCBGACTC-3′) and PolR (5′-ATSGCCATCATYTCRCCGGA-3′) were used to amplify the gene [69]. Although these primers will not extend to amplify all types of diazotrophs (e.g., cyanobacteria and cluster IV nifH), they should amplify the range of organisms expected to exist in soybean nodules. Primers had the Illumina adapters ligated to them where the following PCR conditions were used: 95 °C for 3 min, followed by 30 cycles of 95 °C for 30 sec, 59 °C for 30 sec, 72 °C for 30 sec, and a final 72 °C for 2 min.

Reads were filtered, trimmed, and merged as above except for a minimum sequence quality threshold of 25 and a minimum merged read length of 125 bases. Further processing was conducted through VSEARCH [70]. Reads were dereplicated and then clustered to OTUs (cluster_fast) using a sequence similarity threshold of 0.97. Potential chimeric clusters were detected and removed using the UCHIME algorithm’s [71] de novo method implementation in VSEARCH. This was followed by removal of singletons and OTUs having a single read in all libraries. Finally, nucleotide sequences were corrected by FrameBot for the existence of frameshifts using a minimum protein identity threshold of 0.8 and a minimum length of 41 amino acid residues [72]. A representative selection of sequences (Additional file 3), obtained from Zehr lab’s *nifH* database, was used for downstream taxonomic and phylogenetic analyses [19]. Taxonomy was assigned using the RDP classifier at a confidence level of 0.75 through QIIME’s interface [73]. The top 53 abundant *Bradyrhizobium* OTUs in addition to most abundant OTUs from other phyla accounting for 95% of total reads were selected. They were aligned with the representative *nifH* database using muscle v3.8.31 [74]. Trimal v1.4 was used to clean alignments and remove gaps at a threshold of 0.7[75]. To estimate an evolutionary model, jModeltest2 was used to select from 84 models using the AIC, BIC, and AICc criteria [76]. All criteria chose the GTR+G model. A phylogenetic tree was generated in RaXML using the GTRGAMMA model and 1000 non-parametric bootstraps [77]. The tree was annotated and visualized in iTOL v3[78].

### Amino acid profiling

Amino acid profiling was used as a means to assess a functional fingerprint independent of PICRUSt. Nodules collected from soybean roots were rinsed in a sterile 0.9% NaCl solution three times in order to remove surface soil on the nodules. The rinsed nodules were freeze-dried for 24 h. Dry nodules were transferred to 2-ml centrifuge tube with a sterile 4 mm glass bead. The nodule tubes were submerged in liquid nitrogen and shaken on geno-grinder at 237 rpm for 30 sec. Twenty milligrams of ground nodules were placed into 2 ml-centrifuge tube. One ml of 0.9% NaCl and internal standard, α-Aminobutyric acid (AABA), were added into the centrifuge tube. The mixture was sonicated for 15 min and centrifuged at room temperature for 15 min at 4000×*g*. After centrifugation, the supernatant was collected in a 15-ml conical tube. One milligram of 0.9% NaCl was added to the centrifuge tube and repeat sonication and centrifugation twice, and a total of 3 ml of supernatant was collected. 0.9% NaCl was added to bring the final volume to 5 ml. The supernatant pool was vortexed to mix well and filtered through 0.22-μm PVDF membrane syringe filter. An aliquot of 50 μL of the filtrate was taken for centrifugal vacuum drying [79].

The dry aliquots of filtrates from soil and nodule extractants were reconstituted in 10 μl 0.05 M HCl and finally derivatized using the AccQ FluorTM reagent kit (Fluorescent 6-Aminoquinoly-N-Hydroxysuccinimidyl Carbamate derivatizing reagent; Waters Co. Cat# WAT052880) following the standard protocol from Bosch et al. and Hou et al. [80, 81].

Chromatographic separation on the HPLC 1260 Infinity system (Agilent Technologies, USA) was carried out on a reversed phase column (Waters X-Terra MS C18, 3.5 μm, 2.1×150 mm). The mobile phase consisted of (A) an aqueous solution containing 140 mM of sodium acetate, 17 mM of triethylamine (TEA; Fisher Chemical, Cat# O4884100), and 0.1% (g/L, *w*/*v*) disodium dihydrogen ethylenediaminetetraacetate dihydrate (EDTA-2Na 2H2O; Sigma, CAS# 6381-92-6), pH 5.05, adjusted with phosphoric acid solution, and (B) acetonitrile (ACN; HPLC grade, Fisher Chemical, Cat# A998-1): ultrapure water (60:40, *v*/*v*). The gradient conditions were 0–17 min 100–93% A, 17–21 min 93–90% A, 21–30 min 90–70% A, 30–35 min 70% A, 35–36 min 70–0% A, and then hold for 4 min before restoring to the initial composition at 40.5 min, with the final composition kept for 9 min. The column was thermostated at 50 °C and operated at a flow rate of 0.35 ml/min. The sample injection volume was 5 μL. The analytes detection was carried out using a fluorescence detector (λex = 250 nm and λem = 395 nm). Hydrolyzable amino acids in the samples were qualified and quantified by comparison with amino acid standard solutions. Each amino acid standard solution contained 23 amino acids including 20 protein amino acids: alanine (Ala), arginine (Arg), aspartic acid (Asp), asparagine (Asn), cystine (Cys–Cys; more stable form in oxidative condition than monomer cysteine), glutamic acid (Glu), glutamine (Gln), glycine (Gly), histidine (His), isoleucine (Ile), leucine (Leu), lysine (Lys), methionine (Met), phenylalanine (Phe), proline (Pro), serine (Ser), threonine (Thr), tyrosine (Tyr), tryptophan (Trp), and valine (Val) and non-protein amino acids: citrulline (Cit), γ-Aminobutyric acid (GABA), and ornithine (Orn). An exception is noted for Cys-cys (cysteine dimer). Cysteine (Cys) was only detected in a form of a dimer due to the formation of disulfide bond between two Cys under oxidized conditions. Raw data are provided in Additional file 4.

For statistical analysis, amino acid data were not retrieved for six samples, and they were subsequently excluded from analysis. Asparagine was detected in only three samples, which was below permissible detection limits, and was also eliminated from analysis. Outlier readings were set to NA. Bray-Curtis distances of the Wisconsin transformation of square root values were used as a conservative approach to compare molar percent amino acid. Non-metric multidimensional scaling (NMDS) was performed using the vegan package in R. MRPP was used to test for the effects of irrigation and cultivar on the log-transformed values from the amino acid profiles.

### Integrated network analysis

To have an integrated analysis, all data sources were combined as followed. The top abundant nifH clusters above 0.1%, all 16S rRNA taxa at the family level, and all amino acids profiled were selected together. A meta-analysis of the Spearman correlations between the different amino acids, nifH clusters, and families was conducted based on the irrigated and not-irrigated treatments following the Transkingdom protocol [82]. For generating the network, an edge was considered significant if it passed the principles of causality [83], had the same sign with the p-value of 0.3 in the meta-analysis, and FDR corrected *p* value of 0.1. The network was visualized using Cystoscope v.3.5.1[84].

### Plant physical and physiological measurements

Several measurements were taken for the soybeans throughout the lifetime of plants in the field. Measurements were taken from plants in the right and left rows and averaged per sample in plot. Chlorophyll content was measured using a SPAD-502 chlorophyll meter (Minolta Co. limited, Japan). Gas exchange through stomatal conductance was measured from the bottom side of the leaf using a Leaf Porometer Model SC-1 (Decagon Devices Inc., Pullman, WA, USA). Leaf surface area was measured using an LI 3100. Deer and insect damage were surveyed and reported on a scale from 1 to 5 with one being minimal damage and 5 being severe damage. The prevalent pest causing damage to the plants was the Japanese beetles (*Popillia japonica*). Nodule size index was calculated by multiplying the nodule weight in grams by 1000 and dividing the product by the nodule count. For yield calculation, the inner two rows were harvested using a Wintersteiger classic combine. Seeds were sieved from the soil, cleaned from other debris, and dried to uniform moisture. Weight was adjusted to 13% moisture content and reported in bushes per acre.

## Additional files

Additional file 1: **Figure S1**. Taxonomic summary of the cumulative relative abundance of nodule bacterial communities. **Figure S2**. Rarefaction plots of the alpha diversities of each of the irrigation treatments based on *nifH* OTUs. **Figure S3**. Boxplot showing variability between the nine cultivars based on multivariate beta dispersions. **Figure S4**. Geographic distribution of the overall relative abundance of *Bradyrhizobium* species isolated from fields in soybean main growing regions in the USA. **Figure S5**. The scatter plot shows no clear relationship or trends between yield and the alpha diversities on the family rank (a–b) and the OTU level (c–d). **Figure S6**. The scatter plot shows weak to moderate relationship between yield and top 6 OTUs in both the bacterial communities based on the 16S rRNA gene (a–f) and the diazotroph population based on the *nifH* gene (g–l). **Figure S7**. Variation in microbial communities affects metabolism and nitrogen resource allocation in the nodules as predicted by PICRUSt. **Figure S8**. Boxplot shows nodule size variation along the nine cultivars. **Table S1**. List of significantly different metabolic pathways between soybean cultivars as predicted by PICRUSt. **Table S2**. Vector fitting *R*^2^ scores of the amino acids’ vectors on the NMDS of the amino acids profile. Significantly enriched amino acids in each treatment are highlighted in bold. **Table S3**. Weekly climate data summary from Kentland’s farm, obtained from the onsite weather station for the duration of growing season in 2014. **Table S4**. The response of the bulk soil water content to the irrigation, cultivar and their interaction as determined by a split-plot analysis of variance. **Table S5**. The response of stomatal conductance to the irrigation, cultivar and their interaction as determined by a split-plot analysis of variance. (PDF 893 kb)

Additional file 2: **Figure S10.** Overview of the study design and workflow. (PDF 92 kb)

Additional file 3: **Dataset 1**. Subset of the *nifH* database that was used to assign taxonomy and build the phylogenetic tree. (XLSX 47 kb)

Additional file 4: **Dataset 2.** Raw data of amino acid profiling. (CSV 14 kb)

## Abbreviations

NMDS: Non-metric multidimensional scaling
PCoA: Principle coordinate analysis
