# Supplementary material for: Unprecedented bacterial community richness in soybean nodules vary with cultivar and water status
Source: Microbiome. 2019 Apr 16;7:63. doi: 10.1186/s40168-019-0676-8 (PMC6469096; doi:10.1186/s40168-019-0676-8)
Supplement: Supplementary file 1 — Figure S1. Taxonomic summary of the cumulative relative abundance of nodule bacterial communities. Figure S2. Rarefaction plots of the alpha diversities of each of the irrigation treatments based on nifH OTUs. Figure S3. Boxplot showing variability between the nine cultivars based on multivariate beta dispersions. Figure S4. Geographic distribution of the overall relative abundance of Bradyrhizobium species isolated from fields in soybean main growing regions in the USA. Figure S5. The scatter plot shows no clear relationship or trends between yield and the alpha diversities on the family rank (a–b) and the OTU level (c–d). Figure S6. The scatter plot shows weak to moderate relationship between yield and top 6 OTUs in both the bacterial communities based on the 16S rRNA gene (a–f) and the diazotroph population based on the nifH gene (g–l). Figure S7. Variation in microbial communities affects metabolism and nitrogen resource allocation in the nodules as predicted by PICRUSt. Figure S8. Boxplot shows nodule size variation along the nine cultivars. Table S1. List of significantly different metabolic pathways between soybean cultivars as predicted by PICRUSt. Table S2. Vector fitting R2 scores of the amino acids’ vectors on the NMDS of the amino acids profile. Significantly enriched amino acids in each treatment are highlighted in bold. Table S3. Weekly climate data summary from Kentland’s farm, obtained from the onsite weather station for the duration of growing season in 2014. Table S4. The response of the bulk soil water content to the irrigation, cultivar and their interaction as determined by a split-plot analysis of variance. Table S5. The response of stomatal conductance to the irrigation, cultivar and their interaction as determined by a split-plot analysis of variance. (PDF 893 kb) [file 40168_2019_676_MOESM1_ESM.pdf]

ADDITIONAL FILE 1

Unprecedented Bacterial Family-level Community Richness in Soybean Nodules Vary Broadly With Cultivar and Water Status

Hazem Sharaf[a], Richard R. Rodrigues[a,*], Jinyoung Moon[b], Bo Zhang[b], Kerri Mills[b], Mark A. Williams[a,b#]

[a]Interdisciplinary PhD Program in Genetics, Bioinformatics, and Computational Biology, Virginia Polytechnic Institute and State University, Blacksburg, VA, USA

[b]School of Plant and Environmental Sciences, Virginia Polytechnic Institute and State University, Blacksburg, VA, USA

*Present address: Department of Pharmaceutical Sciences, Oregon State University, Corvallis, OR, USA.

#corresponding author: Mark A. Williams, email: markwill@vt.edu

Table of Contents:

Table of Contents	Page 1
Figure S1	Page 2
Figure S2	Page 4
Figure S3	Page 6
Figure S4	Page 8
Figure S5	Page 10
Figure S6	Page 12
Figure S7	Page 14
Figure S8	Page 16
Table S1	Page 18
Table S2	Page 21
Table S3	Page 22
Table S4	Page 23
Table S5	Page 23
References	Page 24

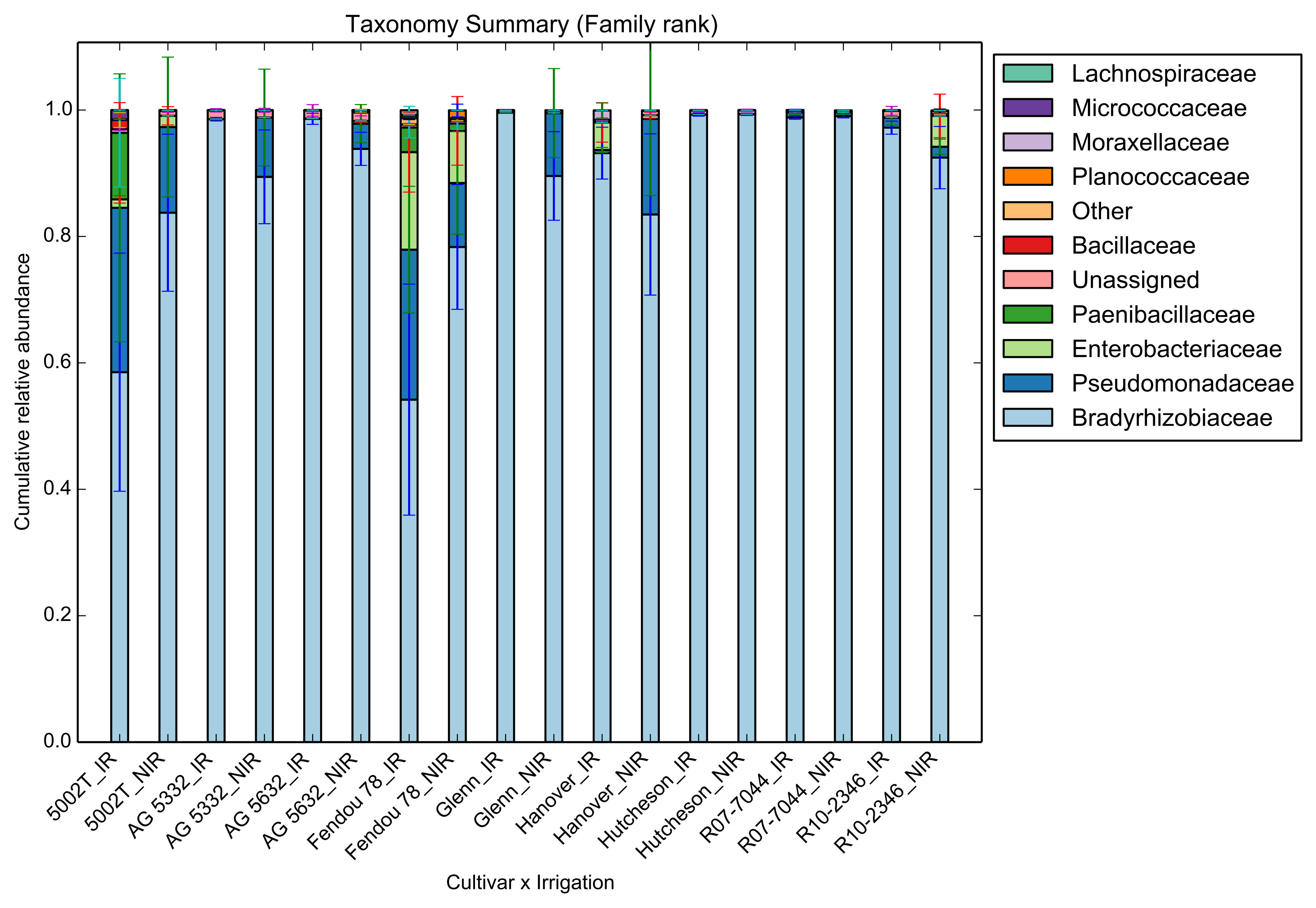

Taxonomy Summary (Family rank)
Cumulative relative abundance
0.0
0.2
0.4
0.6
0.8
1.0
5002T_IR
5002T_NIR
AG 5332_IR
AG 5332_NIR
AG 5632_IR
AG 5632_NIR
Fendou 78_IR
Fendou 78_NIR
Glenn_IR
Glenn_NIR
Hanover_IR
Hanover_NIR
Hutcheson_IR
Hutcheson_NIR
R07-7044_IR
R07-7044_NIR
R10-2346_IR
R10-2346_NIR
Cultivar x Irrigation
Lachnospiraceae
Micrococcaceae
Moraxellaceae
Planococcaceae
Other
Bacillaceae
Unassigned
Paenibacillaceae
Enterobacteriaceae
Pseudomonadaceae
Bradyrhizobiaceae

Figure S1. Taxonomic summary of cumulative relative abundance of nodule bacterial communities. This is a breakdown of Figure 1, illustrating the irrigation effect within cultivar. Taxonomies are shown at the family rank, where the top 10 most abundant families are shown in addition to the summation of the remaining families. Error bars indicate the standard error. 5002T, Hanover and Glenn show major changes in bacterial communities between irrigation treatments.

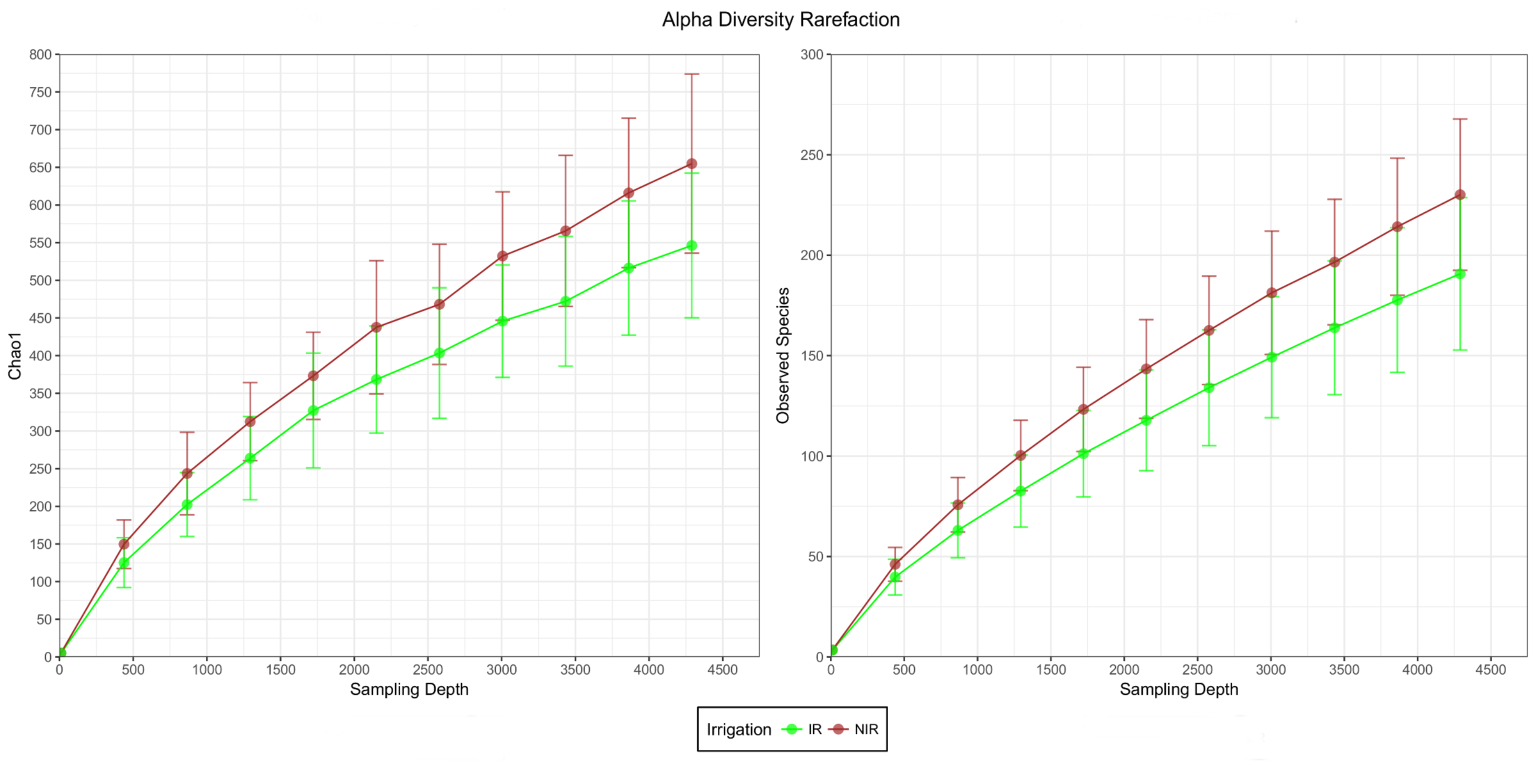

Alpha Diversity Rarefaction
Chao1
Sampling Depth
Observed Species
Sampling Depth
Irrigation
IR
NIR

Figure S2. Rarefaction plots of the alpha diversities of each of irrigation treatments based on *nifH* OTUs. Two alpha diversities indices were used to show the species richness, **(a)** chao1 and **(b)** observed species. Plots were generated from 10 samplings as indicated on the x-axis. The not-irrigated treatment is significantly more diverse than the irrigated treatment for both indices (p-value < 0.01).

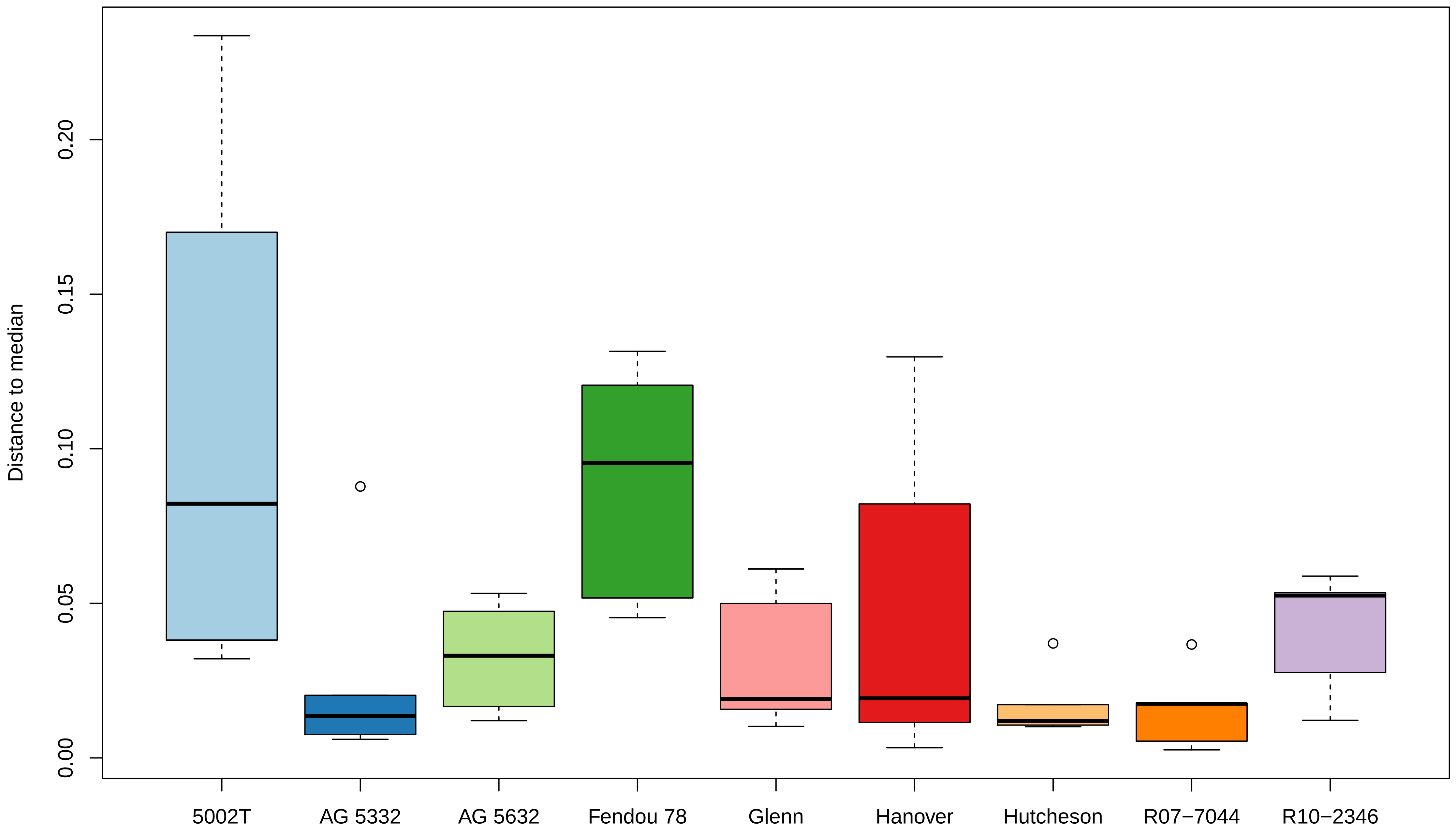
Distance to median
0.00
0.05
0.10
0.15
0.20
5002T
AG 5332
AG 5632
Fendou 78
Glenn
Hanover
Hutcheson
R07−7044
R10−2346

Figure S3. Boxplot showing variability between the nine cultivars based on multivariate beta dispersions. This is a supporting measure of beta diversity between the cultivars. The y-axis shows the average distances of the samples from the overall cultivar median. It is clear that 5002T has the highest variation and deviation from other samples, followed by Fendou-78. This has been supported by Tukey's HSD analysis of the dispersion values.

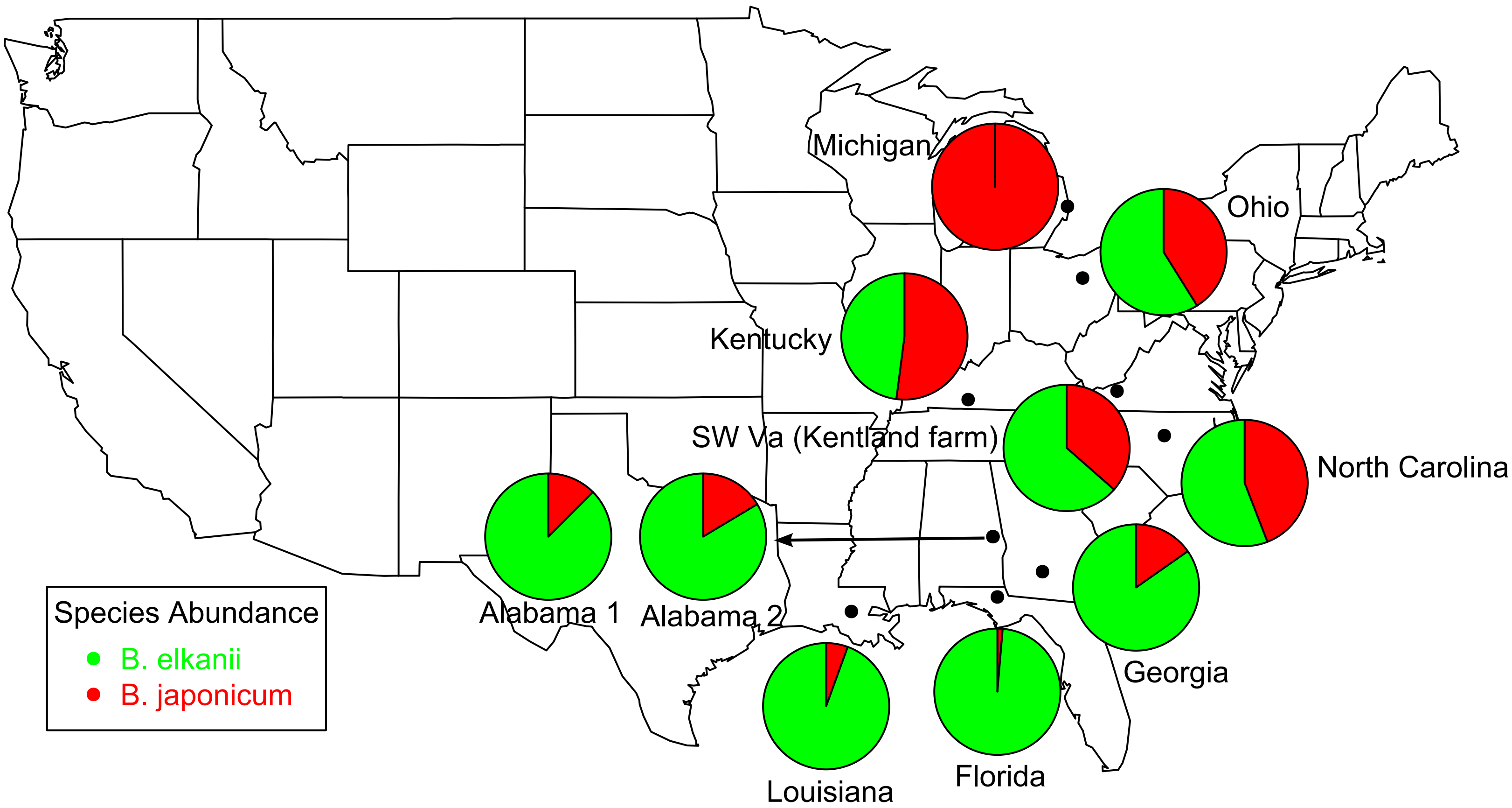

Michigan
Ohio
Kentucky
SW Va (Kentland farm)
North Carolina
Alabama 1
Alabama 2
Georgia
Louisiana
Florida
Species Abundance
B. elkanii
B. japonicum

Figure S4. Geographic distribution of the overall relative abundance of *Bradyrhizobium* species isolated from fields in soybean main growing regions in the United States.
There is a clear north to south variation between *B. japonicum* in the north and *B. elkanii* in the south. The arrow points to the location of the site of this study in southwest Virginia. Data about other locations were obtained from Shiro. et al (1).

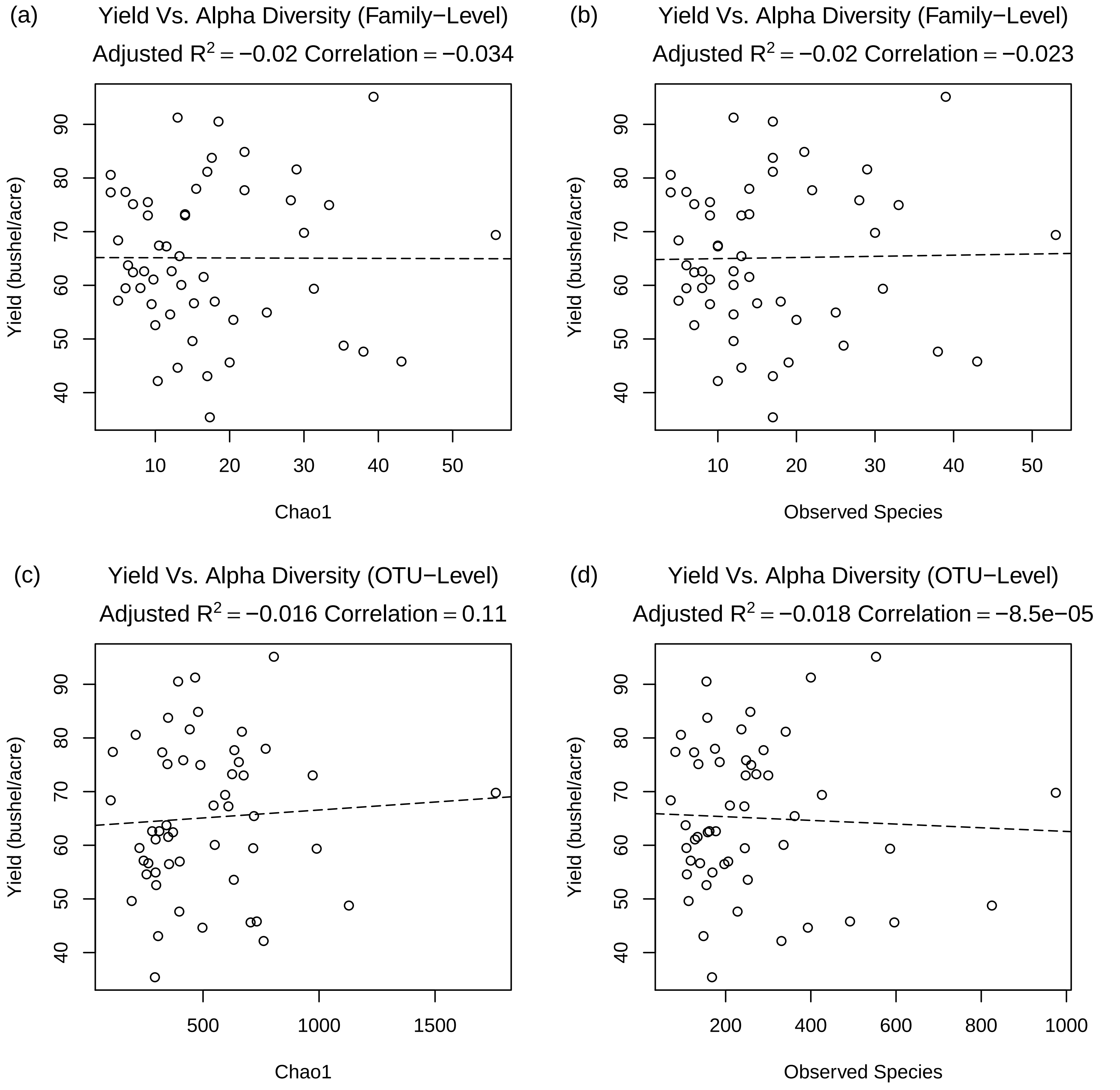
(a)
Yield Vs. Alpha Diversity (Family−Level)
Adjusted R² = −0.02 Correlation = −0.034
Yield (bushel/acre)
Chao1
(b)
Yield Vs. Alpha Diversity (Family−Level)
Adjusted R² = −0.02 Correlation = −0.023
Yield (bushel/acre)
Observed Species
(c)
Yield Vs. Alpha Diversity (OTU−Level)
Adjusted R² = −0.016 Correlation = 0.11
Yield (bushel/acre)
Chao1
(d)
Yield Vs. Alpha Diversity (OTU−Level)
Adjusted R² = −0.018 Correlation = −8.5e−05
Yield (bushel/acre)
Observed Species

Figure S5. The scatter plot shows no clear relationship or trends between yield and the alpha diversities on the family rank (a-b) and the OTU level (c-d). The top of each panel shows adjusted regression model coefficient of determination, and the spearman correlation.

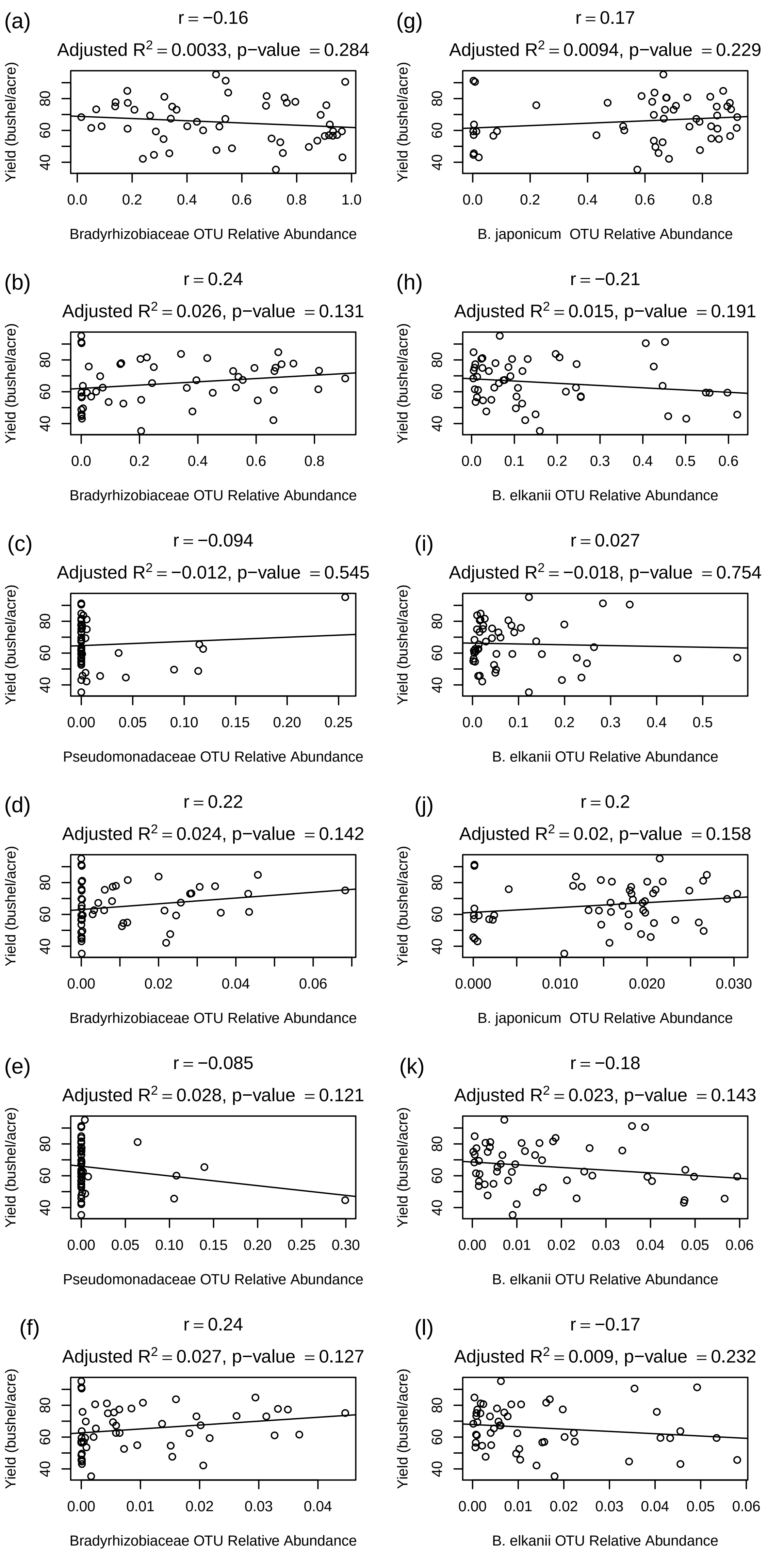

(a)
r = −0.16
Adjusted R² = 0.0033, p−value = 0.284
Yield (bushel/acre)
Bradyrhizobiaceae OTU Relative Abundance
(g)
r = 0.17
Adjusted R² = 0.0094, p−value = 0.229
Yield (bushel/acre)
B. japonicum OTU Relative Abundance
(b)
r = 0.24
Adjusted R² = 0.026, p−value = 0.131
Yield (bushel/acre)
Bradyrhizobiaceae OTU Relative Abundance
(h)
r = −0.21
Adjusted R² = 0.015, p−value = 0.191
Yield (bushel/acre)
B. elkanii OTU Relative Abundance
(c)
r = −0.094
Adjusted R² = −0.012, p−value = 0.545
Yield (bushel/acre)
Pseudomonadaceae OTU Relative Abundance
(i)
r = 0.027
Adjusted R² = −0.018, p−value = 0.754
Yield (bushel/acre)
B. elkanii OTU Relative Abundance
(d)
r = 0.22
Adjusted R² = 0.024, p−value = 0.142
Yield (bushel/acre)
Bradyrhizobiaceae OTU Relative Abundance
(j)
r = 0.2
Adjusted R² = 0.02, p−value = 0.158
Yield (bushel/acre)
B. japonicum OTU Relative Abundance
(e)
r = −0.085
Adjusted R² = 0.028, p−value = 0.121
Yield (bushel/acre)
Pseudomonadaceae OTU Relative Abundance
(k)
r = −0.18
Adjusted R² = 0.023, p−value = 0.143
Yield (bushel/acre)
B. elkanii OTU Relative Abundance
(f)
r = 0.24
Adjusted R² = 0.027, p−value = 0.127
Yield (bushel/acre)
Bradyrhizobiaceae OTU Relative Abundance
(l)
r = −0.17
Adjusted R² = 0.009, p−value = 0.232
Yield (bushel/acre)
B. elkanii OTU Relative Abundance

Figure S6. The scatter plot shows weak to moderate relationship between yield and top 6 OTUs in both the bacterial communities based on the 16S rRNA gene (a-f) and the diazotroph population based on the *nifH* gene (g-l). The top of each panel shows adjusted regression model coefficient of determination, the p-value of the regression and the spearman correlation. The x-axis label includes the taxonomy classification most likely associated at the class level for 16S-based OTU, and at the species level for the nifH-based OTU.

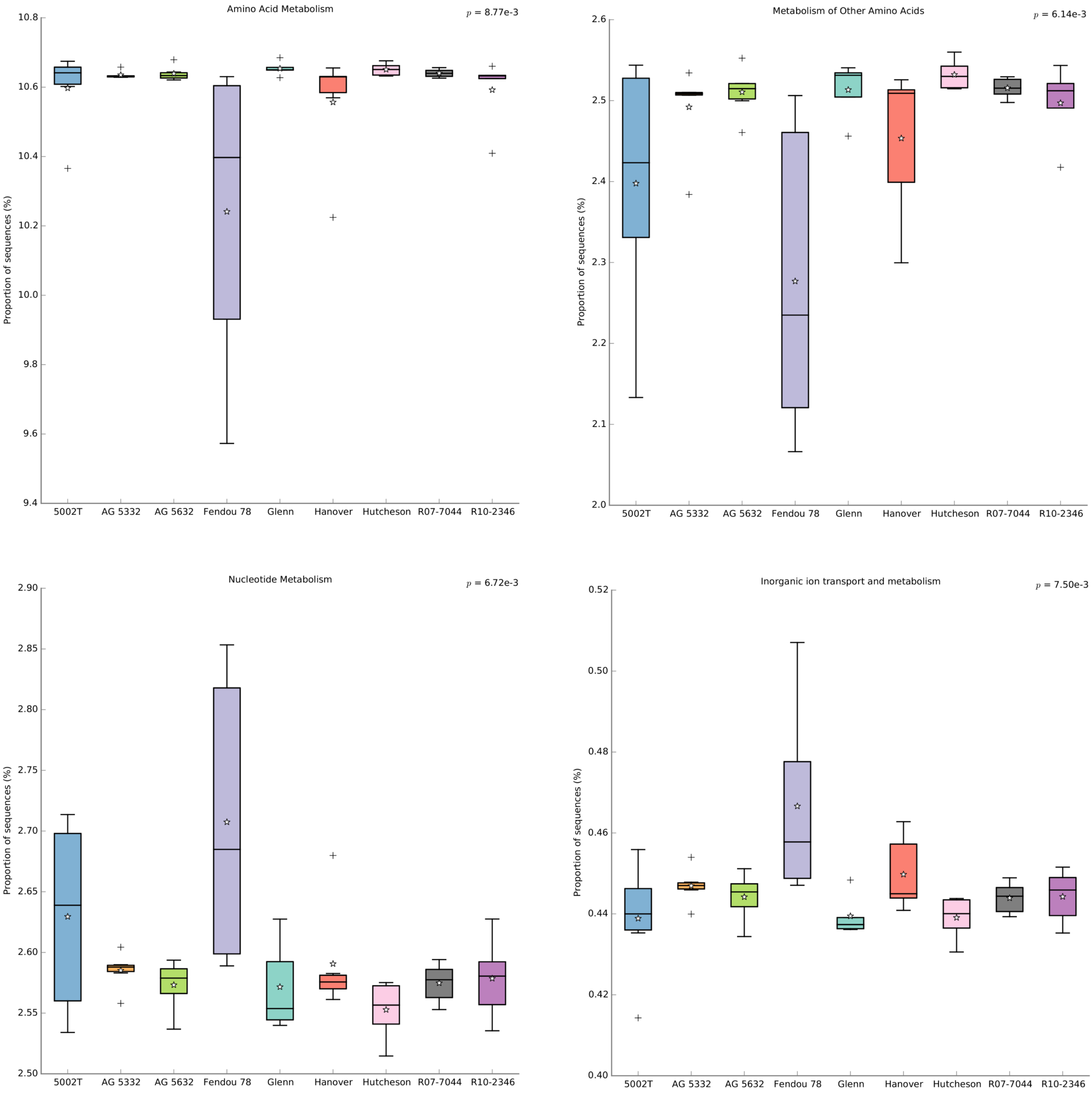

Amino Acid Metabolism
p = 8.77e-3
Metabolism of Other Amino Acids
p = 6.14e-3
Nucleotide Metabolism
p = 6.72e-3
Inorganic ion transport and metabolism
p = 7.50e-3
Proportion of sequences (%)
5002T
AG 5332
AG 5632
Fendou 78
Glenn
Hanover
Hutcheson
R07-7044
R10-2346

Figure S7. Variation in microbial communities affect metabolism and nitrogen resource allocation in the nodules as predicted by PICRUSt. Level-2 KEGG pathways are shown at percentage abundance of all reads after being normalized based on 16S copy numbers as estimated by PICRUSt. (**a**) Amino acid metabolism, (**b**) Metabolism of other amino acids, (**c**) Nucleotide metabolism and (**d**) Inorganic ion transport and metabolism were significantly different between the different cultivars as per Benjamini Hochberg corrected p-values (q-value <0.05).

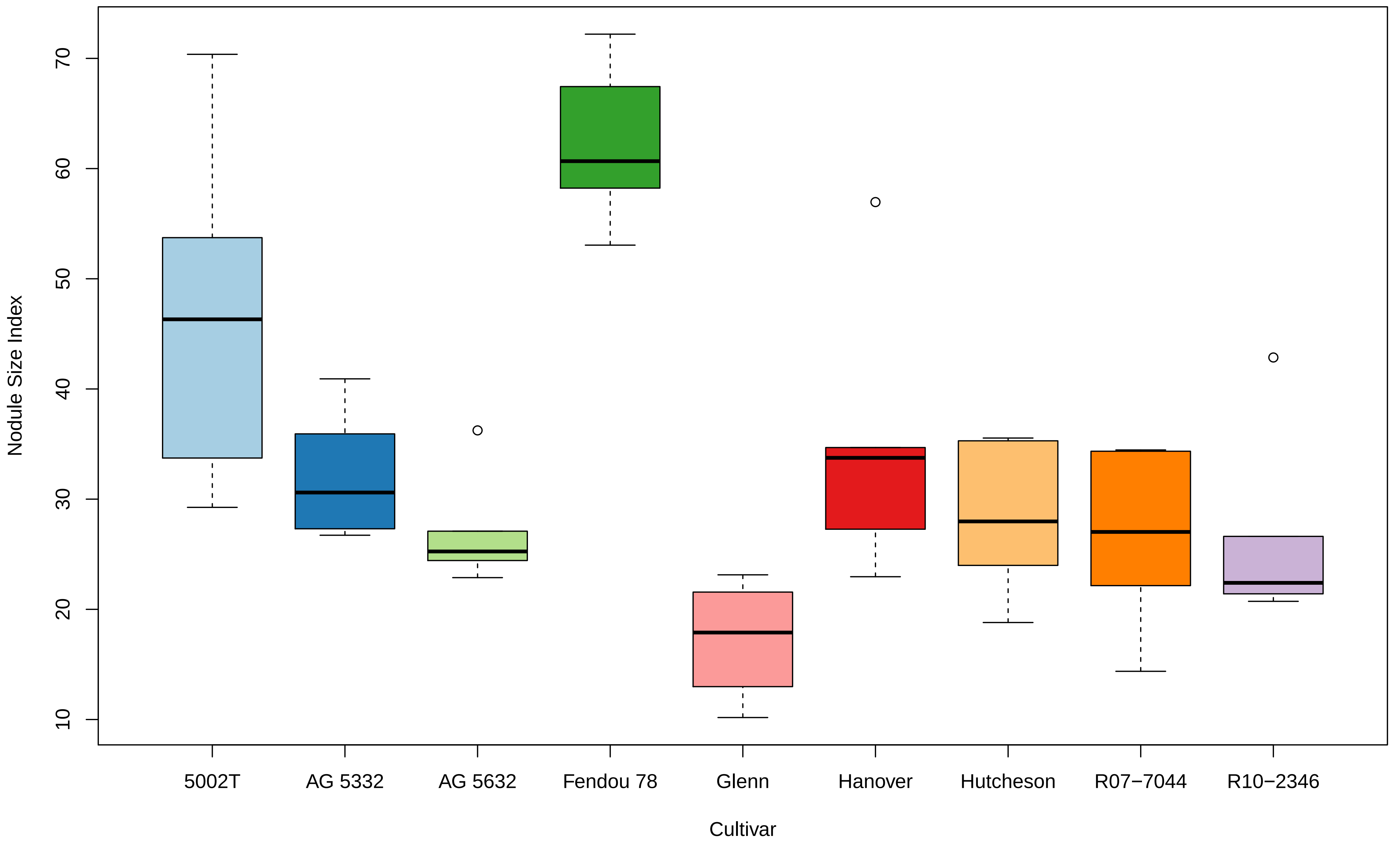
Nodule Size Index
10
20
30
40
50
60
70
5002T
AG 5332
AG 5632
Fendou 78
Glenn
Hanover
Hutcheson
R07−7044
R10−2346
Cultivar

Figure S8. Boxplot shows nodule size variation along the nine cultivars. Nodule size index was the plant parameter with highest correlation to the variation in the microbial communities as determined by the Mantel test. ANOVA has shown that nodule size is affected by the cultivar type rather the irrigation treatment.

Table S1: List of significantly different metabolic pathways between soybean cultivars as predicted by PICRUSt.

KEGG Level 2	KEGG Level 3
Amino Acid Metabolism	Amino Acid metabolism_Unclassified
	Amino acid related enzymes
	Cysteine and methionine metabolism
	Lysine biosynthesis
	Lysine degradation
	Phenylalanine metabolism
	Phenylalanine, tyrosine and tryptophan biosynthesis
	Tryptophan metabolism
	Tyrosine metabolism
	Valine, leucine and isoleucine degradation
Biosynthesis and biodegradation of secondary metabolites	Biosynthesis and biodegradation of secondary metabolites_Unclassified
Biosynthesis of Other Secondary Metabolites	beta-Lactam resistance
	Flavonoid biosynthesis
	Novobiocin biosynthesis
	Penicillin and cephalosporin biosynthesis
	Stilbenoid, diarylheptanoid and gingerol biosynthesis
Carbohydrate Metabolism	Amino sugar and nucleotide sugar metabolism
	Butanoate metabolism
	Carbohydrate metabolism_Unclassified
	Glyoxylate and dicarboxylate metabolism
	Propanoate metabolism
	Starch and sucrose metabolism
Energy Metabolism	Energy metabolism_Unclassified
	Methane metabolism
	Photosynthesis
	Photosynthesis proteins
Enzyme Families	Peptidases
	Protein kinases
Glycan Biosynthesis and Metabolism	Glycan Biosynthesis and Metabolism_Unclassified
	Glycosaminoglycan degradation
	Glycosphingolipid biosynthesis - globo series
	Glycosyltransferases

	Lipopolysaccharide biosynthesis
	Lipopolysaccharide biosynthesis proteins
	Peptidoglycan biosynthesis
Lipid Metabolism	alpha-Linolenic acid metabolism
	Arachidonic acid metabolism
	Fatty acid biosynthesis
	Fatty acid metabolism
	Glycerolipid metabolism
	Glycerophospholipid metabolism
	Linoleic acid metabolism
	Lipid biosynthesis proteins
	Steroid hormone biosynthesis
	Synthesis and degradation of ketone bodies
Metabolism of Cofactors and Vitamins	Lipoic acid metabolism
	Nicotinate and nicotinamide metabolism
	One carbon pool by folate
	Retinol metabolism
	Riboflavin metabolism
	Ubiquinone and other terpenoid-quinone biosynthesis
	Vitamin B6 metabolism
Metabolism of Other Amino Acids	CyanoAmino Acid metabolism
	D-Arginine and D-ornithine metabolism
	D-Glutamine and D-glutamate metabolism
	Glutathione metabolism
	Phosphonate and phosphinate metabolism
	Taurine and hypotaurine metabolism
Metabolism of Terpenoids and Polyketides	Carotenoid biosynthesis
	Geraniol degradation
	Limonene and pinene degradation
	Terpenoid backbone biosynthesis
	Zeatin biosynthesis
Nucleotide Metabolism	Nucleotide Metabolism_Unclassified
	Pyrimidine metabolism
Others	Others_Unclassified
Xenobiotics Biodegradation and Metabolism	Aminobenzoate degradation
	Atrazine degradation
	Bisphenol degradation

Caprolactam degradation
Chloroalkane and chloroalkene degradation
Chlorocyclohexane and chlorobenzene degradation
Drug metabolism - cytochrome P450
Ethylbenzene degradation
Fluorobenzoate degradation
Metabolism of xenobiotics by cytochrome P450
Naphthalene degradation
Nitrotoluene degradation
Polycyclic aromatic hydrocarbon degradation
Styrene degradation
Toluene degradation

Table S2: Vector fitting R2 scores of the amino acids' vectors on the NMDS of the amino acids profile. Significantly enriched amino acids in each treatment are highlighted in bold.

Amino	R2	P-value (FDR)	Treatment
**Asp**	**0.7273**	0.002442	**Irrigated**
Ser	0.0755	0.224407	
Glu	0.0585	0.254745	
Gly	0.4398	0.002442	
His	0.4098	0.002442	
Gln	0.137	0.053006	
Arg	0.0669	0.243956	
**Cit**	**0.6364**	**0.002442**	**Not-Irrigated**
**Thr**	**0.4028**	**0.002442**	**Not-Irrigated**
Ala	0.2146	0.010989	
GABA	0.1538	0.03663	
Pro	0.2432	0.005994	
**Tyr**	**0.2664**	**0.004396**	**Not-Irrigated**
**Cys**	**0.4093**	**0.002442**	**Irrigated**
**Val**	**0.4449**	**0.002442**	**Not-Irrigated**
Met	0.0977	0.131868	
Orn	0.1379	0.053006	
Ile	0.2135	0.013525	
**Lys**	**0.5772**	**0.002442**	**Not-Irrigated**
**Leu**	**0.1953**	**0.015699**	**Not-Irrigated**
Phe	0.0654	0.254745	
**Trp**	**0.5721**	**0.002442**	**Irrigated**

Table S3. Weekly climate data summary from Kentland's farm, obtained from the onsite weather station for the duration of growing season in 2014.

Month/Week	Precipitation (mm)	Air temperature °C	Soil temperature °C
May W1	0	16.22733871	15.55790323
May W2	53.086	18.39801075	18.45849462
May W3	0	15.04068817	16.73983871
May W4	26.162	19.36464324	18.72756757
June W1	11.938	19.02027778	19.32077778
June W2	15.748	21.10066667	20.20833333
June W3	2.286	22.78227778	20.88816667
June W4	6.604	22.51566667	22.22688889
July W1	0	21.92392473	23.02795699
July W2	30.988	21.90091398	23.3672043
July W3	10.414	20.63467742	22.06811828
July W4	31.242	20.67387097	22.90672043
August W1	17.018	19.6194086	22.13758065
August W2	93.726	18.59586022	20.8155914
August W3	37.338	21.81467742	22.74623656
August W4	0.254	20.4788172	22.46564516
September W1	46.736	21.81855556	22.40883333
September W2	44.196	18.68344444	20.73638889
September W3	6.35	16.26166667	18.97388889
September W4	0.762	15.76216667	17.6255
October W1	43.942	13.27206452	16.30548387
October W2	56.388	14.80610753	16.27951613
October W3	2.032	10.67683871	14.18155914
October W4	9.144	10.19126882	12.6038172

Table S4. The response of the bulk soil water content to the irrigation, cultivar and their interaction as determined by a split-plot analysis of variance.

	Df	Sum Sq	Mean Sq	F value	Pr(>F)	
Replicate	2	2.953	1.476	1.3387	0.42758	
**Irrigation**	**1**	**73.844**	**73.844**	**66.9558**	**0.01461**	*
Error A	2	2.206	1.103			
Cultivar	8	46.032	5.754	1.1018	0.39358	
Irrigation:Cultivar	8	13.823	1.728	0.3309	0.94623	
Error B	26	135.778	5.222			

Table S5. The response of stomatal conductance to the irrigation, cultivar and their interaction as determined by a split-plot analysis of variance.

	Df	Sum Sq	Mean Sq	F value	Pr(>F)	
Replicate	2	2266	1133.2	1.2498	0.44448	
**Irrigation**	**1**	**10200**	**10199.8**	**11.249**	**0.07856**	.
Whole plot error	2	1813	906.7			
Error A	8	16217	2027.2	1.039	0.42839	
Irrigation:Cultivar	8	6291	786.4	0.4031	0.91045	
Error B	32	62434	1951.1			

References:

1. Shiro S, Matsuura S, Saiki R, Sigua GC, Yamamoto A, Umehara Y, et al. Genetic Diversity and Geographical Distribution of Indigenous Soybean-Nodulating Bradyrhizobia in the United States. Appl Environ Microbiol [Internet]. 2013;79:3610–8. Available from: http://aem.asm.org/lookup/doi/10.1128/AEM.00236-13
