# Supplementary material for: Unprecedented bacterial community richness in soybean nodules vary with cultivar and water status
Source: Microbiome. 2019 Apr 16;7:63. doi: 10.1186/s40168-019-0676-8 (PMC6469096; doi:10.1186/s40168-019-0676-8)
Supplement: Supplementary file 2 — Figure S10. Overview of the study design and workflow. (PDF 92 kb) [file 40168_2019_676_MOESM2_ESM.pdf]

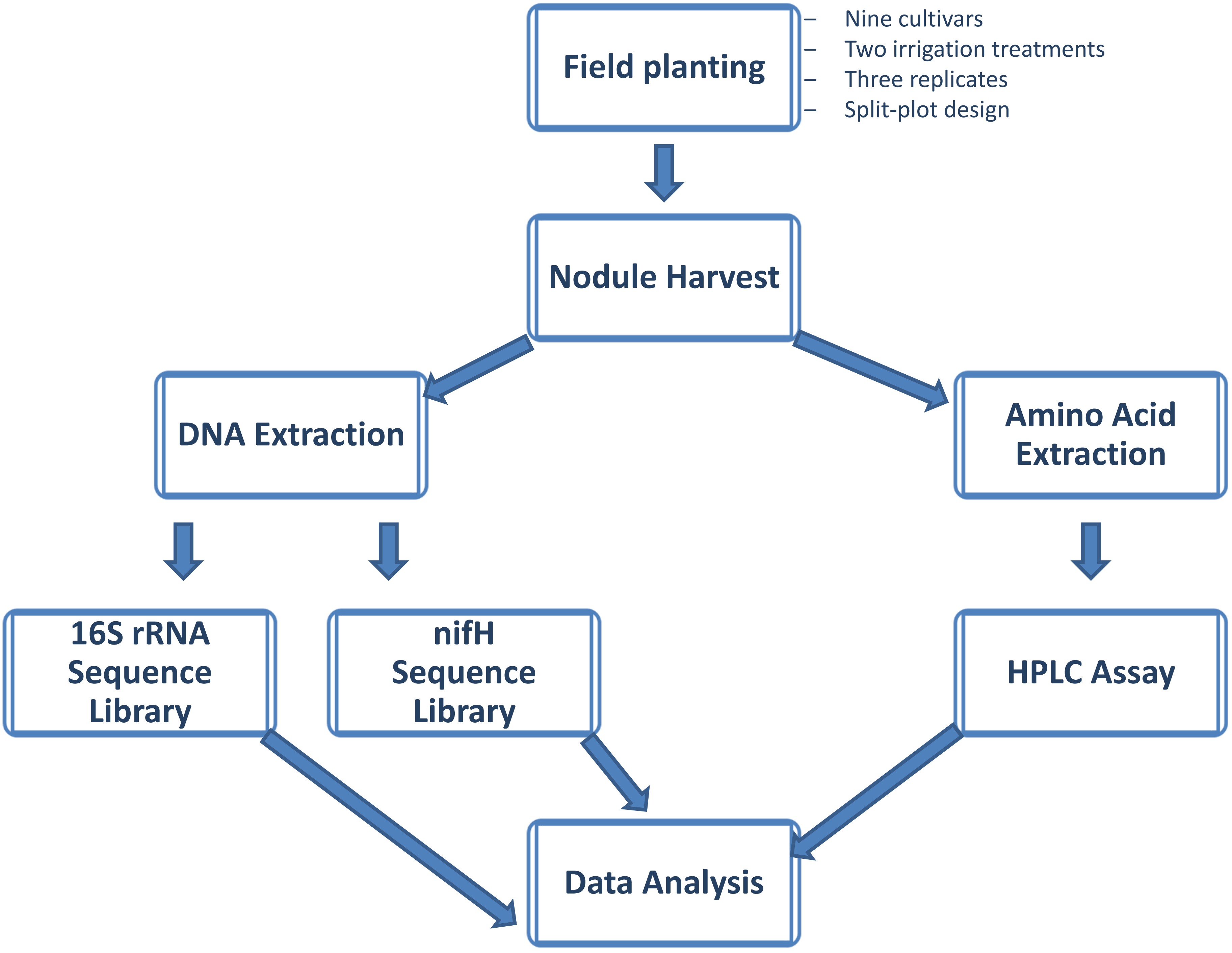
Field planting
– Nine cultivars
– Two irrigation treatments
– Three replicates
– Split-plot design
Nodule Harvest
DNA Extraction
Amino Acid Extraction
16S rRNA Sequence Library
nifH Sequence Library
HPLC Assay
Data Analysis
